# Anti-Inflammatory Effects of Serotonin Receptor and Transient Receptor Potential Channel Ligands in Human Small Intestinal Epithelial Cells

**DOI:** 10.3390/cimb45080427

**Published:** 2023-08-15

**Authors:** Gregory Ian Robinson, Dongping Li, Bo Wang, Yeva Zahoruiko, Marta Gerasymchuk, Darryl Hudson, Olga Kovalchuk, Igor Kovalchuk

**Affiliations:** 1 Department of Biological Sciences, University of Lethbridge, Lethbridge, AB T1K 3M4, Canada; g.robinson@uleth.ca (G.I.R.); marta.gerasymchuk@uleth.ca (M.G.); 2 GoodCap Pharmaceuticals, Calgary, AB T2P 0R3, Canada

**Keywords:** psilocybin, ketanserin, 4-AcO-DMT, curcumin, eugenol, capsaicin, serotonin receptor ligands, transient receptor potential channel ligands, inflammation, small intestine

## Abstract

Intestinal inflammation and dysbiosis can lead to inflammatory bowel diseases (IBD) and systemic inflammation, affecting multiple organs. Developing novel anti-inflammatory therapeutics is crucial for preventing IBD progression. Serotonin receptor type 2A (5-HT2A) ligands, including psilocybin (Psi), 4-Acetoxy-N,N-dimethyltryptamine (4-AcO-DMT), and ketanserin (Ket), along with transient receptor potential (TRP) channel ligands like capsaicin (Cap), curcumin (Cur), and eugenol (Eug), show promise as anti-inflammatory agents. In this study, we investigated the cytotoxic and anti-inflammatory effects of Psi, 4-AcO-DMT, Ket, Cap, Cur, and Eug on human small intestinal epithelial cells (HSEIC). HSEIC were exposed to tumor necrosis factor (TNF)-α and interferon (IFN)-γ for 24 h to induce an inflammatory response, followed by treatment with each compound at varying doses (0–800 μM) for 24 to 96 h. The cytotoxicity was assessed using the 3-(4,5-dimethylthiazol-2-yl)-2,5-diphenyltetrazolium bromide (MTT) assay and protein expression by Western blot (WB) analysis. As single treatments, Psi (40 μM), Cur (0.5 μM), and Eug (50 μM) significantly reduced COX-2 levels without cytotoxic effects. When combined, Psi (40 μM) and Cur (0.5 μM) exhibited synergy, resulting in a substantial decrease in COX-2 protein levels (−28× fold change), although the reduction in IL-6 was less pronounced (−1.6× fold change). Psi (20 μM) and Eug (25 μM) demonstrated the most favorable outcomes, with significant decreases in COX-2 (−19× fold change) and IL-6 (−10× fold change) protein levels. Moreover, the combination of Psi and Eug did not induce cytotoxic effects in vitro at any tested doses. This study is the first to explore the anti-inflammatory potential of psilocybin and 4-AcO-DMT in the intestines while highlighting the potential for synergy between the 5-HT2A and TRP channel ligands, specifically Psi and Eug, in alleviating the TNF-α/IFN-γ-induced inflammatory response in HSEIC. Further investigations should evaluate if the Psi and Eug combination has the therapeutic potential to treat IBD in vivo.

## 1. Introduction

Inflammation in the small intestine plays a critical role in maintaining the health and integrity of the gut lining, which is essential for proper nutrient absorption and digestion. The inflammatory response helps to remove harmful bacteria, viruses, and toxins that may be present in the gut and helps to repair damaged tissue. However, chronic or excessive inflammation can have negative effects on the small intestine, leading to inflammatory bowel disease (IBD), whereby the body’s immune system mistakenly attacks the intestinal lining, causing chronic inflammation and damage to the tissue [1]. In 2019, there were 4.9 million cases of IBD globally [2], while the rates are continuing to rise [3]. While there are some treatments for IBD, including medications and lifestyle changes, there are no known cures [4].

Psilocybin is a hallucinogenic substance found in certain species of mushrooms that has been studied for its potential therapeutic effects. While its effects are not fully understood, some research suggests that psilocybin may have anti-inflammatory effects [5]. When consumed, it is converted into psilocin in the body, which then acts on various serotonin receptors in the brain. Psilocin primarily acts on the 5-HT2A receptors [6], which are a subtype of serotonin receptors that are involved in regulating mood, perception, and cognition [7]. 5-HT2A receptors have been found on T cells, B cells, and monocytes, and their activation has been shown to modulate immune function and cytokine production [5]. Psilocin also has an affinity for other serotonin receptor subtypes, including 5-HT2B [6]. These receptors are found on T cells, B cells, and macrophages and have been shown to modulate the proliferation and differentiation of immune cells, cytokine production, and immune cell migration. 5-HT2B receptors also play a role in regulating the immune response during inflammation and autoimmune diseases [8].

One study found that psilocybin reduced inflammation in the brains of rats by decreasing the activity of certain immune cells called microglia [8]. Recently, the oral administration of psilocybin and eugenol in vivo ameliorated LPS-induced inflammation within the brain [9]. While more research is needed to fully understand the anti-inflammatory effects of psilocybin, these findings suggest that it may have potential as a therapeutic agent for a range of conditions characterized by inflammation in the body or brain.

4-acetoxy-N,N-dimethyltryptamine (4-AcO-DMT) is a synthetic hallucinogen similar to psilocybin. 4-AcO-DMT is the O-acetylated version of psilocin, which is deacylated within the liver to produce psilocin. Since both psilocybin and 4-AcO-DMT convert into psilocin within the body, 4-AcO-DMT has been suggested as a replacement for psilocybin, as 4-AcO-DMT is much easier and cheaper to produce if similar effects are reproduced by 4-AcO-DMT [10]. While there is growing evidence of the anti-inflammatory effects of psilocybin and other serotonin receptor ligands [5], no research has been performed on the anti-inflammatory effects of 4-AcO-DMT.

While ketanserin is not commonly used as an anti-inflammatory agent due to its known adverse effects, some studies have suggested that it may have anti-inflammatory effects. Ketanserin, a selective serotonin receptor antagonist, has been shown to attenuate colitis, whereby ketanserin treatment prevented neutrophil recruitment, inflammatory cytokine production, and apoptosis [11]. In addition, ketanserin can decrease M2 macrophage polarization and migration, decrease nuclear factor kappa B (NF-κB) activation, and ameliorate perturbations in the structure of the intestinal mucosa induced by dextran sodium sulphate [12]. While more research is needed to fully understand the anti-inflammatory effects of ketanserin, the available evidence suggests that ketanserin has potential to aid IBD.

Curcumin is a natural polyphenol found in the turmeric root, which is commonly used as a spice in Indian cuisine and in traditional medicine. Research has shown that curcumin has potent anti-inflammatory effects by inhibiting several proinflammatory signaling pathways, including toll-like receptor 4 (TLR4)/NF-κB signaling, which plays a crucial role in regulating the immune response and inflammation [13]. While the mechanism of action is not fully understood, curcumin has been shown to inhibit the activation of TRPV1, which can lead to a reduction in inflammation [14]. Additionally, curcumin has been shown to decrease the production of proinflammatory cytokines, such as interleukin-6 (IL-6) and tumor necrosis factor alpha (TNF-α), which are involved in inflammatory responses in the intestine [14]. Due to studies showing promising anti-inflammatory effects of curcumin [15] and an acceptable daily intake of up to 3 mg/kg of the body weight, as recognized by the Food Agriculture Organization, World Health Organization, and European Food Safety Authority [16], further studies should examine curcumin’s efficacy as a novel anti-inflammatory agent.

Eugenol is a natural compound found in various plants such as clove, basil, and cinnamon. It has been shown to possess anti-inflammatory effects in several studies, but its effects specifically on the intestine are not well understood. In 2020, eugenol was shown to ameliorate LPS-induced inflammation in porcine intestinal epithelial cells [17]. More recently, eugenol has been shown to maintain the intestinal barrier integrity and possess anti-inflammatory properties in transmissible gastroenteritis virus-induced inflammation in piglets [18]. While there is a growing amount of literature on the anti-inflammatory effects of eugenol, some research has suggested that combining eugenol with other compounds may provide synergistic effects [9,19]. More research is still required to determine if combinations of eugenol with other compounds can have synergistic effects within the intestines.

Capsaicin, the compound responsible for the spicy taste of chili peppers, has been shown to have a wide range of anti-inflammatory effects [20]. Capsaicin is known to activate the transient receptor potential vanilloid 1 (TRPV1), which is found in immune cells; however, TRPV1-independent anti-inflammatory mechanisms have been demonstrated [21]. Prolonged TRPV1 agonism by capsaicin can have an anti-inflammatory effect by inhibiting NF-κB and decreasing the release of inflammatory cytokines [22]. In addition, capsaicin has been shown to alleviate microbial dysbiosis and improve intestinal barrier function [20]. Furthermore, capsaicin can help alleviate high-fat diet-induced chronic inflammation within mice through improved gut microbiota function [23]. While capsaicin has been shown to have beneficial anti-inflammatory effects on colitis in animal models, no research has investigated the anti-inflammatory effects of capsaicin in the small intestine [22].

The aim of this study was to test the efficacy of serotonin receptor 2 ligands psilocybin, 4-AcO-DMT, and ketanserin, as well as transient receptor potential channel ligands capsaicin, curcumin, and eugenol, to reduce the inflammatory response in HSEIC treated with TNF-α/INF-γ. The synergism between drug classes was tested, as the doses of hallucinogenic drugs can be reduced if synergistic effects exist, thereby enabling the anti-inflammatory effects of psychedelics to be recapitulated without unwanted hallucinogenic effects. In addition, we aimed to test the cytotoxic effects of these compounds to accurately guide what doses should be utilized. Due to differences in the binding affinity and biased agonism [24], we hypothesize that select combinations will be more efficacious in reducing the inflammatory response within HSEIC.

## 2. Materials and Methods

### 2.1. Cell Culture and Maintenance

Healthy human small intestine epithelial cells (HSIEC) were obtained from Cell Biologics (#H-6051, Chicago, IL, USA). Cells were cultured in a complete human epithelial cell medium/w kit (#H6621, Cell Biologics, Chicago, IL, USA). All cells were incubated at 37 °C in a humidified atmosphere of 5% CO_2_ within our humidified Forma Steri-Cycle CO_2_ incubator (Thermo Fisher Scientific, Waltham, MA, USA) in our BSL-2 laboratory at the University of Lethbridge. Cell culture media were replenished every three days while being subcultured at 80% confluency. HSIEC were treated with the mycoplasma removal reagent BM-Cyclin (#10799050001, Roche, Mississauga, ON, Canada) to prevent mycoplasma contamination. HSIEC grown to 80% confluency were treated with 10 ng/mL TNF-α/IFN-γ (Sigma, Markham, ON, Canada) for 24 h.

### 2.2. Methyl Thiazole Tetrazolium (MTT) Cytotoxicity Assay

The cytotoxicity of psilocybin (Sigma, Markham, ON, Canada), 4-AcO-DMT (ChemLogix, Burnaby, BC, Canada), ketanserin (TCI America, Portland, OR, USA), capsaicin (Sigma, Markham, ON, Canada), curcumin (Sigma, Markham, ON, Canada), and eugenol (Sigma, Markham, ON, Canada) were measured using the microculture methyl thiazole tetrazolium (MTT, 3-[4,5-dimethylthiazol-2-yl]-2,5-diphenyltetrazolium bromide; thiazolyl blue) colourimetric metabolic activity assay. MTT was performed utilizing a cell proliferation kit (#11465007001, Roche, Mississauga, ON, Canada), according to the manufacturer’s instructions. Once the HSIEC had grown to 80% confluency, 3 × 10^3^ cells/well were replated in 96-well plates. Twenty-four hours after incubation in 96-well plates, the cells were treated with the indicated concentration of psilocybin, 4-AcO-DMT, ketanserin, capsaicin, curcumin, and eugenol, individually or in combination. Each compound, individually and in combination, was tested at 0, 24, 48, 72, and 96 h. Psilocybin and 4-AcO-DMT were tested at the following concentrations: 0, 5, 10, 20, 40, 80, and 160 μM. Ketanserin and curcumin were tested at the following concentrations: 0, 1, 5, 10, 20, 40, and 80 μM. Capsaicin was tested at the following concentrations: 0, 0.5, 5, 10, 50, 100, and 200 μM. Eugenol was tested at the following concentrations: 0, 10, 50, 100, 200, 400, and 800 μM. DMSO and/or ethanol dissolved into the media served as the vehicle control. The spectrophotometric absorbance of the samples was measured at 595 nm using a microtiter plate reader (FLUOstar Omega, BMG LABTECH, Cary, NC, USA).

### 2.3. Protein Extraction and Quantification

After treatment, the HSIEC were harvested and rinsed with ice-cold PBS and scraped off the plate with a RIPA buffer. The mixture was centrifuged 1600× *g* for five mins, and the supernatant was discarded. Pellets were washed in ice-cold PBS and solubilized in the RIPA lysis buffer. Whole cellular protein lysate was sonicated using a Braunsonic model 1510 sonicator (B. Braun, Melsungen, Germany) operating at an 80% sonication capacity. Lysates were centrifuged for 12,000× *g* for 10 min. The supernatant was collected and stored at −80 °C for further use. To quantify the protein concentrations, the Bradford protein assay was performed via a NanoDrop 2000/2000c Spectrophotometer (Thermo Fisher Scientific, Wilmington, DE, USA).

### 2.4. Western Immunoblotting

Protein (60–100 μg per sample) was electrophoresed on 8% or 10% SDS-PAGE and electrophoretically transferred to a PVDF membrane (Amersham Hybond™-P, GE Healthcare, Arlington Heights, IL, USA) at 4 °C for 1.5 h. Unaltered PVDF membranes were stained with Coomassie blue (Bio-Rad, Hercules, CA, USA) to confirm equal protein loading. Blots were incubated for 1 h with 5% nonfat dry milk to block nonspecific binding sites and subsequently incubated at 4 °C overnight with a 1:200 to 1:1000 dilution of polyclonal/monoclonal antibodies against IL-6 (Santa Cruz Biotechnology, Dallas, TX, USA) and COX-2 (Abcam, Cambridge, UK). Immunoreactivity was detected using a peroxidase-conjugated antibody and visualized by the ECL Plus Western Blotting Detection System (#GERPN2232, GE Healthcare, Arlington Heights, IL, USA). The blots were stripped before re-probing with an antibody against GAPDH (Abcam, Cambridge, UK). Densitometry of the bands was measured and normalized with GAPDH using NIH ImageJ 1.5.0 (64-bit) software.

### 2.5. Statistical Analysis

Each experiment was repeated a minimum of three times (n = 3). The results are represented as the mean per group with the standard deviation (SD) of the mean. Mean values and SD were calculated, analyzed, and plotted with GraphPad Prism 9.5.1 (GraphPad Software, San Diego, CA, USA). Statistical analyses of all data were performed using one-way ANOVA tests, followed by Dunnett’s post hoc multiple comparison tests. Significance (*p*) was indicated within the figures using the following scale: * *p* < 0.05, ** *p* < 0.01, *** *p* < 0.001, and **** *p* < 0.0001.

## 3. Results

### 3.1. The Cytotoxic Effects of Serotonin Receptor and Transient Receptor Potential Channel Ligands

**Figure 1:**
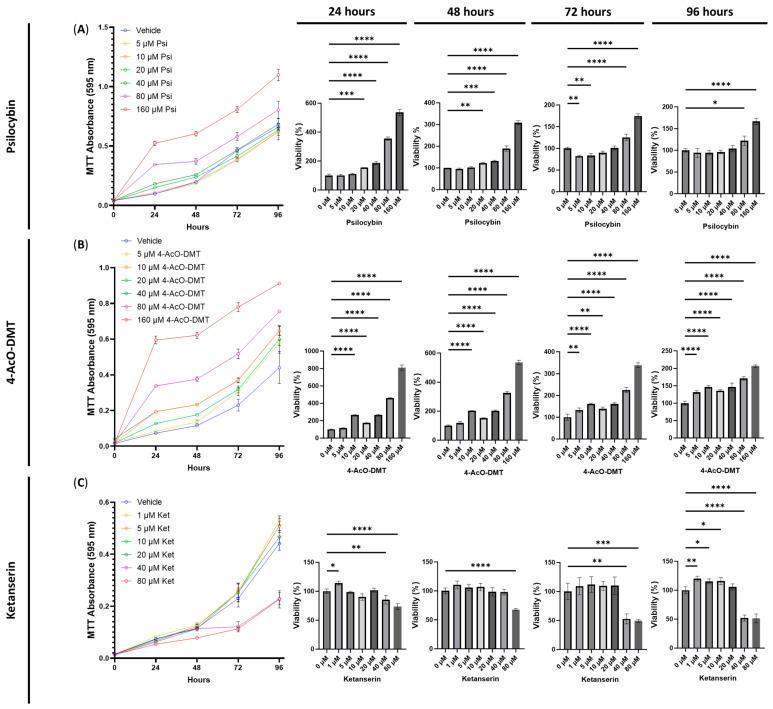
Cell viability of human small intestinal epithelial cells treated with serotonin receptor ligands. Graphs show the cell viability after 24, 48, 72, and 96 h of exposure to (**A**) psilocybin, (**B**) 4-AcO-DMT, or (**C**) ketanserin. Cell viability was quantified by the MTT assay with the absorbance at 595 nm. Data were analyzed with one-way ANOVA followed by a Dunnett’s post hoc test compared to the control group. Bars represent the mean ± SD. Significance is indicated using the following scale: * *p* < 0.05, ** *p* < 0.01, *** *p* < 0.001, and **** *p* < 0.0001. 4-AcO-DMT, 4-acetoxy-N,N-dimethyltryptamine; Ket, ketanserin; Psi, psilocybin.

To assess the cytotoxic effects of serotonin receptor ligands, we conducted experiments using psilocybin, 4-AcO-DMT, and ketanserin at various doses on HSEIC. After 24 h of exposure, psilocybin significantly increased the number of viable cells at doses of 20 (*p* < 0.001), 40, 80, and 160 μM (*p* < 0.0001, Figure 1A). Similarly, after 48 h, psilocybin increased the cell viability at 20 (*p* < 0.01), 40 (*p* < 0.001), 80, and 160 μM (*p* < 0.0001, Figure 1A). At the 72 h mark, the 5 and 10 μM doses significantly decreased the cell viability (*p* < 0.01), while the 80 and 160 μM doses increased the cell viability (*p* < 0.0001, Figure 1A). Finally, exposure to 80 (*p* < 0.05) and 160 μM (*p* < 0.0001) of psilocybin for 96 h significantly increased the cell viability. Throughout the entire experiment, the only cytotoxic effects observed were at 72 h with the 5 and 10 μM doses of psilocybin (Figure 1A).

Next, we examined the cytotoxicity of 4-AcO-DMT. Both at 24 and 48 h, doses of 10, 20, 40, 80, and 160 μM significantly increased the cell viability (*p* < 0.0001, Figure 1B). After 72 h, 5 (*p* < 0.01), 10 (*p* < 0.0001), 20 (*p* < 0.01), 40, 80, and 100 μM (*p* < 0.0001) significantly increased the cell viability (Figure 1B). Similarly, after 96 h of exposure to 4-AcO-DMT, all doses significantly increased (*p* < 0.0001) the cell viability (Figure 1B). Throughout all doses and time points, 4-AcO-DMT did not exhibit cytotoxic effects.

Lastly, we assessed the cytotoxicity of ketanserin. Although ketanserin demonstrated significantly increased cell viability at 1 μM after 24 h (*p* < 0.05) and at 1 (*p* < 0.01), 5 (*p* < 0.05), and 10 μM after 96 h (*p* < 0.05, Figure 1C), it also showed cytotoxic effects. At 80 μM, ketanserin decreased the cell viability after 24 (*p* < 0.0001), 48 (*p* < 0.0001), 72 (*p* < 0.001), and 96 h (*p* < 0.0001, Figure 1C). Additionally, a dose of 40 μM significantly reduced cell viability after 24 (*p* < 0.01), 72 (*p* < 0.01), and 96 h (*p* < 0.0001, Figure 1C).

**Figure 2:**
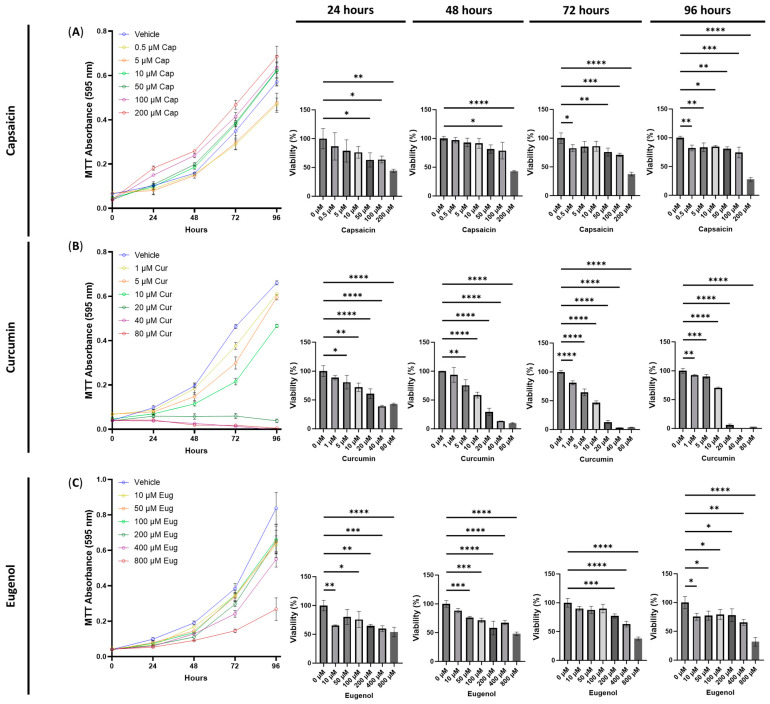
Cell viability of human small intestinal epithelial cells treated with transient receptor potential channel ligands. Graphs show the cell viability after 24, 48, 72, and 96 h of exposure to (**A**) capsaicin, (**B**) curcumin, or (**C**) eugenol. Cell viability was quantified by the MTT assay with the absorbance at 595 nm. Data were analyzed with one-way ANOVA followed by a Dunnett’s post hoc test compared to the control group. Bars represent the mean ± SD. Significance is indicated using the following scale: * *p* < 0.05, ** *p* < 0.01, *** *p* < 0.001, and **** *p* < 0.0001. Cap, capsaicin; Cur, curcumin; Eug, eugenol.

To evaluate the cytotoxicity of transient receptor potential channel ligands, including capsaicin, curcumin, and eugenol, we tested a range of relevant doses. For capsaicin, we found that 50 μM after 24 h (*p* < 0.05), 100 μM after 24 and 48 h (*p* < 0.05), and 200 μM after 24 h (*p* < 0.01) and 48 h (*p* < 0.0001) significantly decreased the cell viability (Figure 2A). In contrast, 0.5 μM of capsaicin exposure decreased the cell viability after 72 (*p* < 0.05) and 96 h (*p* < 0.01) compared to the vehicle. In addition, 50 (*p* < 0.01), 100 (*p* < 0.001), and 200 μM (*p* < 0.0001) of capsaicin exposure for 72 h decreased the cell viability compared to the vehicle (Figure 2A). After 96 h of exposure, all doses significantly decreased the cell viability (*p* < 0.05, Figure 2A).

Surprisingly, curcumin demonstrated cytotoxic effects across all doses after 48, 72, and 96 h (*p* < 0.01, Figure 2B). Furthermore, doses of 5 (*p* < 0.05), 10 (*p* < 0.01), 20 (*p* < 0.0001), 40 (*p* < 0.0001), and 80 μM (*p* < 0.0001, Figure 2B) exhibited significantly lower cell viability after 24 h, while 1 μM showed no difference compared to the control. The most substantial and severe decreases in cell viability were observed at 20, 40, and 80 μM, displaying a decreasing MTT absorbance over time (Figure 2B).

Lastly, eugenol demonstrated both short-term and long-term cytotoxic effects (Figure 2C). At 24 h, 10 (*p* < 0.01), 100 (*p* < 0.05), 200 (*p* < 0.01), 400 (*p* < 0.001), and 800 (*p* < 0.0001) μM of eugenol decreased the cell viability, while exposure to 50 (*p* < 0.001), 100 (*p* < 0.001), 200 (*p* < 0.0001), 400 (*p* < 0.0001), and 800 μM (*p* < 0.0001) for 48 h significantly reduced the cell viability (Figure 2C). Similarly, after 72 h of exposure, only 200 (*p* < 0.001), 400 (*p* < 0.0001), and 800 μM (*p* < 0.0001) of eugenol significantly reduced the cell viability compared to the vehicle (Figure 2C). In contrast, all doses reduced the cell viability compared to the vehicle after 96 h of eugenol exposure (*p* < 0.05, Figure 2C).

### 3.2. The Cytotoxic Effects of Combined Treatments of Serotonin Receptor and Transient Receptor Potential Channel Ligands

Next, we conducted experiments to test the combinations of psilocybin, 4-AcO-DMT, or ketanserin with capsaicin, curcumin, or eugenol. Based on the cytotoxicity data presented in Figure 2, we selected doses of 0.5 and 1 μM for both capsaicin and curcumin, while using 25 μM for eugenol. For psilocybin and 4-AcO-DMT, we used doses of 10, 20, and 40 μM, as these doses showed minimal cytotoxicity and did not demonstrate strong proliferative effects (Figure 1). Similarly, for ketanserin, we tested doses of 1, 5, and 10 μM, as these doses had the least impact based on the MTT assays shown in Figure 1.

**Figure 3:**
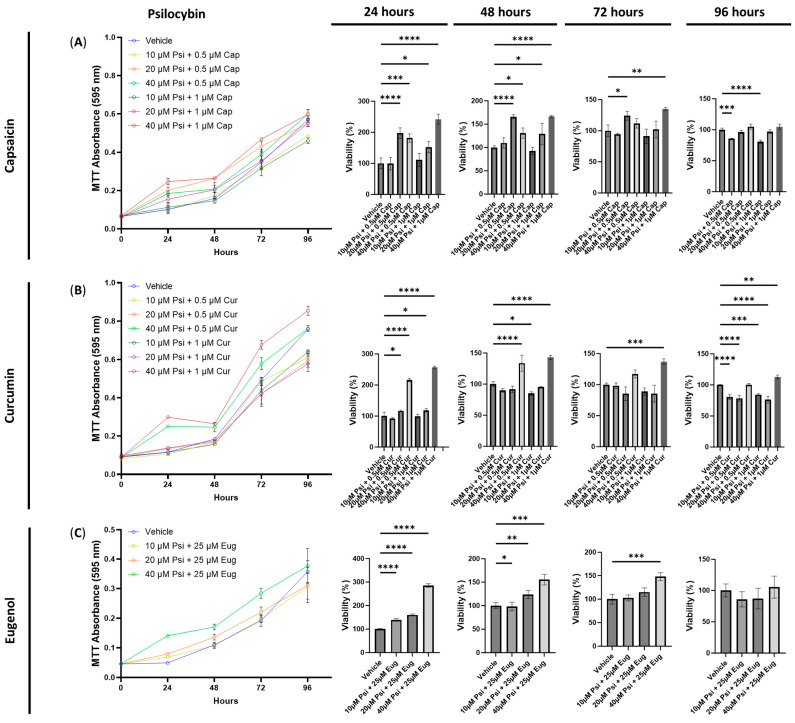
Cell viability of human small intestinal epithelial cells treated with psilocybin combined with transient receptor potential channel ligands. Graphs show the cell viability after 24, 48, 72, and 96 h of exposure to psilocybin combined with (**A**) capsaicin, (**B**) curcumin, or (**C**) eugenol. Cell viability was quantified by the MTT assay with the absorbance at 595 nm. Data were analyzed with one-way ANOVA followed by a Dunnett’s post hoc test compared to the vehicle. Bars represent the mean ± SD. Significance is indicated using the following scale: * *p* < 0.05, ** *p* < 0.01, *** *p* < 0.001, and **** *p* < 0.0001. Cap, capsaicin; Cur, curcumin; Eug, eugenol; Psi, psilocybin.

The combination of psilocybin and capsaicin did not exhibit any cytotoxic effects, except after 96 h for 10 μM of psilocybin combined with either 0.5 μM (*p* < 0.001) or 1 μM of capsaicin (*p* < 0.0001, Figure 3A). In contrast, the combination of psilocybin and capsaicin increased the relative MTT absorbance at 24 and 48 h when using concentrations of 10 and 20 μM of psilocybin combined with 0.5 and 1 μM of capsaicin (*p* < 0.05, Figure 3A). Furthermore, the combination of 20 μM of psilocybin and 0.5 μM of capsaicin (*p* < 0.05), and 40 μM of psilocybin combined with 1 μM of capsaicin (*p* < 0.01), increased the cell viability after 72 h (Figure 3A).

Similarly, combinations of 20 (*p* < 0.05) and 40 μM (*p* < 0.0001) of psilocybin with 0.5 and 1 μM of curcumin increased the cell viability after 24 h, while the combination of 40 μM of psilocybin with either 0.5 or 1 μM of curcumin increased the cell viability after 48 h compared to the control (*p* < 0.0001, Figure 3B). Additionally, 40 μM of psilocybin combined with 1 μM of curcumin significantly increased the cell viability after 72 (*p* < 0.001) and 96 h (*p* < 0.01, Figure 3B). In contrast, the combination of 10 μM of psilocybin and 1 μM of curcumin reduced the cell viability compared to the control after 48 h (*p* < 0.05, Figure 3B). After 96 h, 10 and 20 μM of psilocybin combined with 0.5 μM or 1 μM of curcumin significantly reduced the cell viability (*p* < 0.001, Figure 3B).

Lastly, all combinations of psilocybin and eugenol did not exhibit any cytotoxicity but instead demonstrated significant pro-proliferative effects (Figure 3C). After 24 h, 10, 20, and 40 μM of psilocybin combined with 25 μM of eugenol significantly increased the cell viability compared to the control (*p* < 0.0001, Figure 3C). Similar trends were observed after 48 h for 10 (*p* < 0.05), 20 (*p* < 0.01), and 40 (*p* < 0.001) μM of psilocybin combined with 25 μM of eugenol. After 72 h, 40 μM (*p* < 0.001) of psilocybin combined with 25 μM of eugenol significantly increased the cell viability (Figure 3C). No significant differences were observed at 96 h (Figure 3C).

**Figure 4:**
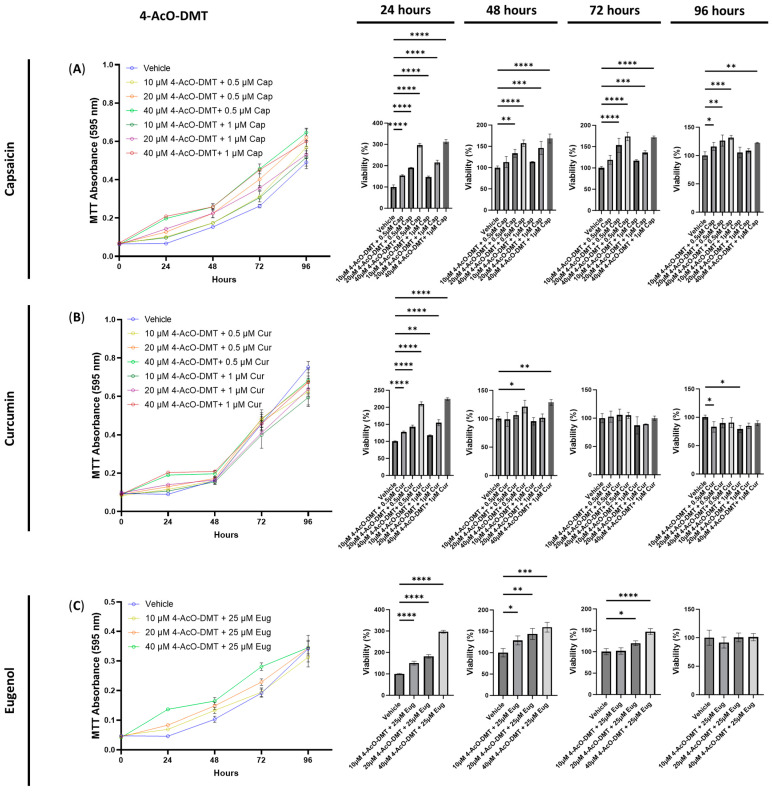
Cell viability of human small intestinal epithelial cells treated with 4-AcO-DMT combined with transient receptor potential channel ligands. Graphs show the cell viability after 24, 48, 72, and 96 h of exposure to 4-AcO-DMT combined with (**A**) capsaicin, (**B**) curcumin, or (**C**) eugenol. Cell viability was quantified by the MTT assay with the absorbance at 595 nm. Data were analyzed with one-way ANOVA followed by a Dunnett’s post hoc test compared to the vehicle. Bars represent the mean ± SD. Significance is indicated using the following scale: * *p* < 0.05, ** *p* < 0.01, *** *p* < 0.001, and **** *p* < 0.0001. 4-AcO-DMT, 4-acetoxy-N,N-dimethyltryptamine; Cap, capsaicin; Cur, curcumin; Eug, eugenol.

Next, we conducted cytotoxicity tests to evaluate the effects of combining 4-AcO-DMT with capsaicin, curcumin, and eugenol. Interestingly, all combinations of 4-AcO-DMT (10, 20, and 40 μM) with capsaicin (0.5 and 1 μM) did not exhibit any cytotoxic effects (Figure 4A). Instead, several combinations resulted in an increased cell viability (Figure 4A). After 24 h of exposure, all combinations significantly increased the cell viability compared to the control (*p* < 0.0001, Figure 4A). Similarly, after 48 h, combinations such as 20 μM of 4-AcO-DMT with either 0.5 (*p* < 0.01) or 1 μM of capsaicin (*p* < 0.001), and 40 μM of 4-AcO-DMT with 0.5 or 1 μM of capsaicin (*p* < 0.0001), increased the cell viability (Figure 4A). These trends continued at 72 h (*p* < 0.001, Figure 4A). At 96 h, combinations of 0.5 μM of capsaicin with 10 (*p* < 0.05), 20 (*p* < 0.01), and 40 μM of 4-AcO-DMT (*p* < 0.001) significantly increased the cell viability (Figure 4A). However, 10 and 20 μM of 4-AcO-DMT combined with 1 μM of capsaicin did not demonstrate significant changes. Nevertheless, 40 μM of 4-AcO-DMT combined with 1 μM of capsaicin did significantly increase the cell viability after 96 h of exposure (*p* < 0.01, Figure 4A).

When examining combinations of 4-AcO-DMT and curcumin, the primary observation was an increase in cell viability or no significant changes. However, after 96 h, cytotoxic effects were observed (Figure 4B). Exposure to 10 μM of 4-AcO-DMT with 0.5 and 1 μM of curcumin significantly decreased the cell viability compared to the control after 96 h (Figure 4B). In contrast, no significant changes were observed at 72 h of exposure, while minimal changes were seen at 48 h, where only 40 μM of 4-AcO-DMT combined with 0.5 (*p* < 0.05) and 1.0 μM (*p* < 0.01) of curcumin showed significant increases in cell viability (Figure 4B). At 24 h of exposure, all combinations of 4-AcO-DMT and curcumin demonstrated significantly higher cell viability compared to the control (*p* < 0.01, Figure 4B).

Finally, we evaluated the cytotoxicity of 4-AcO-DMT combined with eugenol, which did not exhibit any cytotoxic effects. Instead, the cell viability was significantly increased (*p* < 0.0001) after 24 h of exposure to 10, 20, and 40 μM of 4-AcO-DMT combined with 25 μM of eugenol (Figure 4C). Similarly, the combinations of 10 (*p* < 0.05), 20 (*p* < 0.01), and 40 μM (*p* < 0.001) of 4-AcO-DMT with 25 μM of eugenol significantly increased the cell viability after 48 h of exposure. After 72 h, only 20 (*p* < 0.05) and 40 μM (*p* < 0.0001) of 4-AcO-DMT combined with 25 μM of eugenol increased the cell viability (Figure 4C). No significant changes were observed at 96 h (Figure 4C).

**Figure 5:**
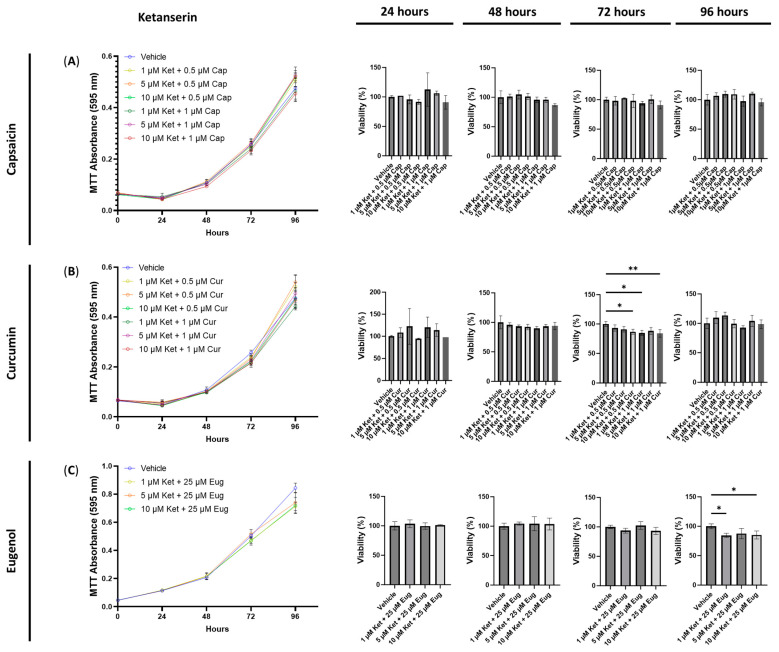
Cell viability of human small intestinal epithelial cells treated with ketanserin combined with transient receptor potential channel ligands. Graphs show the cell viability after 24, 48, 72, and 96 h of exposure to ketanserin combined with (**A**) capsaicin, (**B**) curcumin, or (**C**) eugenol. Cell viability was quantified by the MTT assay with the absorbance at 595 nm. Data were analyzed with one-way ANOVA followed by a Dunnett’s post hoc test compared to the vehicle. Bars represent the mean ± SD. Significance is indicated using the following scale: * *p* < 0.05, ** *p* < 0.01. Cap, capsaicin; Cur, curcumin; Eug, eugenol; Ket, ketanserin.

Next, we examined the cytotoxic effects of combining ketanserin with capsaicin, curcumin, or eugenol. Interestingly, no significant differences in cell viability were observed after 24, 48, 72, or 96 h of exposure to 1, 5, and 10 μM of ketanserin combined with 0.5 or 1 μM of capsaicin (Figure 5A). In contrast, when 10 μM of ketanserin was combined with 0.5 μM of curcumin, a significant decrease in cell viability was observed after 72 h (*p* < 0.05, Figure 5B). Similarly, the combinations of 1 (*p* < 0.05) and 10 μM of ketanserin (*p* < 0.01) with 1 μM of curcumin resulted in a significant decrease in cell viability compared to the control (Figure 5B). Furthermore, the combinations of 1 and 10 μM of ketanserin with 25 μM of eugenol significantly reduced the cell viability after 96 h of exposure (*p* < 0.05, Figure 5C). No other significant differences were observed with any combinations of ketanserin and capsaicin, curcumin, or eugenol (Figure 5).

### 3.3. The Anti-Inflammatory Effects of the Serotonin Receptor or Transient Receptor Potential Channel Ligands

To assess the anti-inflammatory potential of each ligand, we subjected HSIEC to TNF-α/IFN-γ exposure, along with varying concentrations of the ligands. Following the treatment, we collected the cells and quantified COX-2 levels through Western blot analysis.

**Figure 6:**
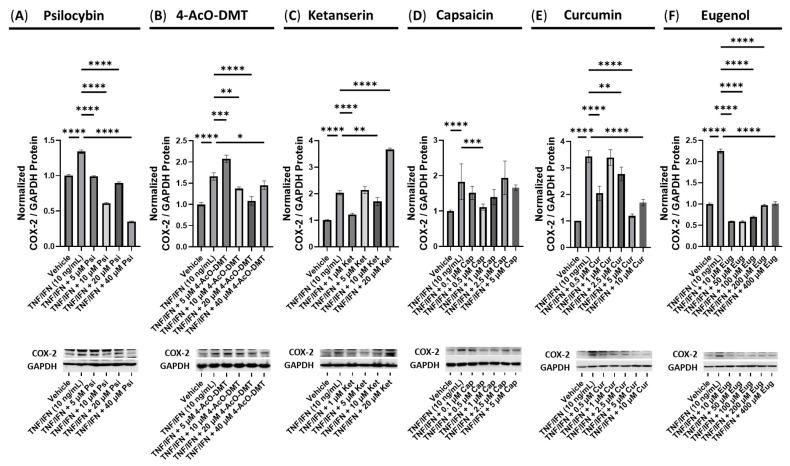
Anti-inflammatory effects of the serotonin receptor or transient receptor potential channel ligand exposure on COX-2 protein expression in human small intestine epithelial cells. Normalized relative densitometry is presented as a ratio of COX-2 to GAPDH for cells exposed to (**A**) psilocybin, (**B**) 4-AcO-DMT, (**C**) ketanserin, (**D**) capsaicin, (**E**) curcumin, and (**F**) eugenol. Cells were exposed to the TNF-α and IFN-γ to induce inflammatory response The original membranes can be seen in the Supplementary Materials. Data were analyzed with an ANOVA followed by a Dunnett’s post hoc multiple comparison test compared to the TNF-α/IFN-γ group. Bars represent the mean ± SD, n = 3. Significance is indicated using the following scale: * *p* < 0.05, ** *p* < 0.01, *** *p* < 0.001, and **** *p* < 0.0001.

Psilocybin demonstrated significant reductions in the normalized COX-2 levels across all concentrations tested (5, 10, 20, and 40 μM) compared to the TNF-α/IFN-γ group (*p* < 0.0001, Figure 6A). Similarly, 4-AcO-DMT showed a significant decrease in the relative COX-2 levels at the concentrations of 10 (*p* < 0.01), 20 (*p* < 0.0001), and 40 μM (*p* < 0.05), while 5 μM significantly increased relative COX-2 levels (*p* < 0.001, Figure 6B) compared to TNF-α/IFN-γ group. In contrast, ketanserin exhibited a limited ability to reduce the inflammatory response, as indicated by the COX-2 levels. Only 1 (*p* < 0.0001) and 10 μM (*p* < 0.01) of ketanserin significantly lowered COX-2 levels (Figure 6C). Strikingly, 20 μM of ketanserin led to a significant increase in the normalized COX-2 levels compared to the TNF-α/IFN-γ group (*p* < 0.0001, Figure 6C).

Regarding capsaicin, a reduction in COX-2 levels was observed only at a dose of 0.5 μM compared to the TNF-α/IFN-γ group (*p* < 0.001, Figure 6D), although both 0.1 and 1 μM showed a trend towards decreased normalized COX-2/GAPDH levels (*p* = N.S., Figure 6D). Curcumin treatment significantly reduced normalized COX-2 levels at concentrations of 0.5 (*p* < 0.0001), 2.5 (*p* < 0.01), 5, and 10 μM (*p* < 0.0001) compared to the TNF-α/IFN-γ group (Figure 6E). In contrast, eugenol exhibited remarkable efficacy in reducing the normalized COX-2 levels at all tested concentrations compared to the TNF-α/IFN-γ group, which was significantly higher than the vehicle (*p* < 0.0001, Figure 6F).

### 3.4. The Anti-Inflammatory Effects of Psilocybin and Transient Receptor Potential Channel Ligands

**Figure 7:**
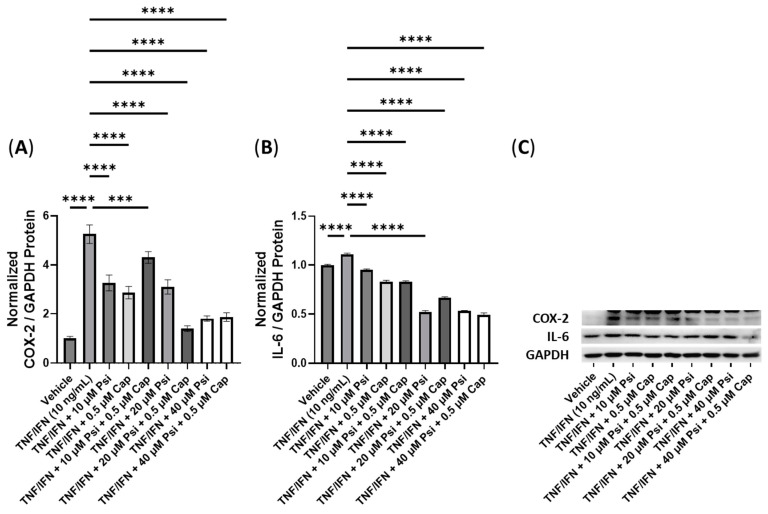
Anti-inflammatory effects of the psilocybin and capsaicin cotreatment on (**A**) COX-2 and (**B**) IL-6 protein expression relative to GAPDH. (**C**) Representative Western blots were analyzed for the relative densitometry of the protein expression and normalized to the control. The original Western blots can be found in the Supplementary Materials. Bars represent the mean ± SD. Data were analyzed with a one-way ANOVA test and a Dunnett’s post hoc multiple comparison test compared to the TFN-α/IFN-γ group. Significance is indicated within the figures using the following scale: *** *p* < 0.001, and **** *p* < 0.0001. Cap, capsaicin; Psi, psilocybin.

In this experiment, we investigated the impact of combinations of psilocybin and capsaicin on the protein levels of COX-2 and IL-6 (Figure 7). Compared to the control, the TNF-α/IFN-γ group exhibited a significant upregulation in COX-2 and IL-6 levels (*p* < 0.0001, Figure 7A,B). Both psilocybin and capsaicin individually effectively reduced COX-2 levels compared to the TNF-α/IFN-γ group (*p* < 0.0001, Figure 7A). Similarly, combinations of psilocybin (20 and 40 μM) and capsaicin (0.5 μM) demonstrated significant reductions in COX-2 levels compared to the TNF-α/IFN-γ group (Figure 7A). However, the combination of 10 μM psilocybin and 0.5 μM capsaicin, although significantly reducing COX-2 levels compared to the TNF-α/IFN-γ group (*p* < 0.001), appeared to be less effective than individual treatments or combinations with higher psilocybin concentrations (Figure 7A). In contrast, all treatment groups exhibited significant reductions in the IL-6 levels compared to the TNF-α/IFN-γ group (*p* < 0.0001), with the highest psilocybin doses showing the greatest differences (Figure 7B). Representative protein bands can be seen in Figure 7C.

**Figure 8:**
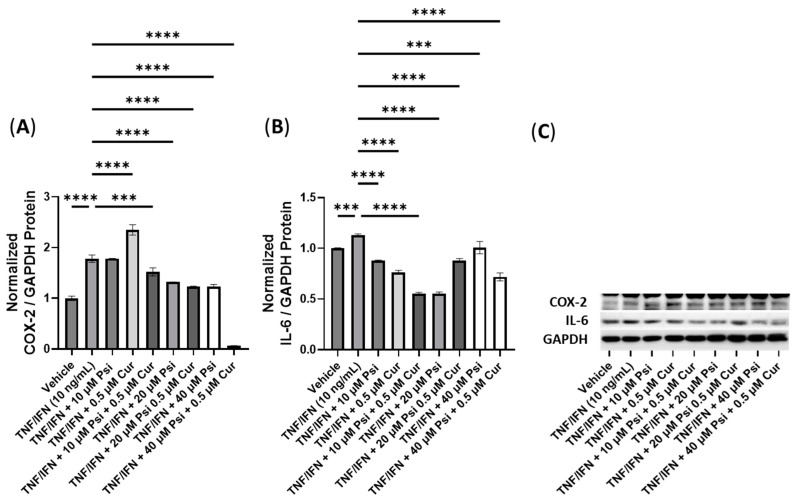
Anti-inflammatory effects of the psilocybin and curcumin cotreatment on (**A**) COX-2 and (**B**) IL-6 protein expression relative to GAPDH. (**C**) Representative Western blots were analyzed for the relative densitometry of the protein expression and normalized to the control. The original Western blots can be found in the Supplementary Materials. Bars represent the mean ± SD. Data were analyzed with a one-way ANOVA test and a Dunnett’s post hoc multiple comparison test compared to the TFN-α/IFN-γ group. Significance is indicated within the figures using the following scale: *** *p* < 0.001, and **** *p* < 0.0001. Cur, curcumin; Psi, psilocybin.

Next, we conducted experiments to assess the effectiveness of combinations of psilocybin and curcumin (Figure 8). The TNF-α/IFN-γ group exhibited significant upregulation in the COX-2 levels compared to the control (*p* < 0.0001, Figure 8A). However, the combination of 10 μM psilocybin and 0.5 μM curcumin significantly reduced the COX-2 levels compared to the TNF-α/IFN-γ group (*p* < 0.001, Figure 8A). Moreover, 20 μM psilocybin combined with 0.5 μM capsaicin, and 40 μM psilocybin all significantly decreased the COX-2 levels compared to the TNF-α/IFN-γ group (*p* < 0.0001, Figure 8A). The most effective treatment in lowering the COX-2 levels compared to the TNF-α/IFN-γ group was the combination of 40 μM psilocybin with 0.5 μM curcumin (*p* < 0.0001, Figure 8A). In contrast, all treatments resulted in decreased IL-6 levels compared to the TNF-α/IFN-γ group (*p* < 0.001), with 20 μM psilocybin and 10 μM psilocybin combined with 0.5 μM curcumin showing the most significant reductions (*p* < 0.0001, Figure 8B). Representative protein bands can be seen in Figure 8C.

**Figure 9:**
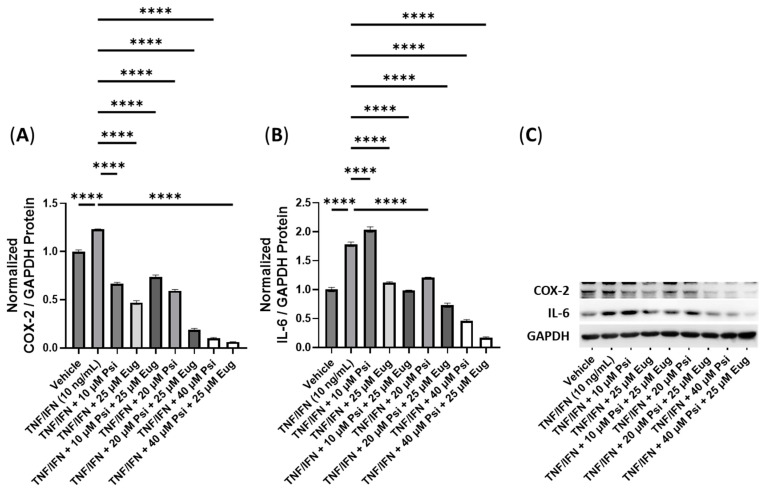
Anti-inflammatory effects of the psilocybin and eugenol cotreatment on (**A**) COX-2 and (**B**) IL-6 protein expression relative to GAPDH. (**C**) Representative Western blots were analyzed for the relative densitometry of the protein expression and normalized to the control. The original Western blots can be found in the Supplementary Materials. Bars represent the mean ± SD. Data were analyzed with a one-way ANOVA test and a Dunnett’s post hoc multiple comparison test compared to the TFN-α/IFN-γ group. Significance is indicated within the figures using the following scale: **** *p* < 0.0001. Eug, eugenol; Psi, psilocybin.

Next, we conducted experiments to assess the efficacy of combining psilocybin with eugenol (Figure 9). The treatment with TNF-α/IFN-γ significantly increased the protein levels of both COX-2 and IL-6 compared to the control (*p* < 0.0001, Figure 9A,B). All treatments involving psilocybin and/or eugenol significantly decreased the COX-2 levels compared to the TNF-α/IFN-γ group (*p* < 0.0001, Figure 9A). In contrast, all treatment groups showed significant downregulation in the IL-6 levels (*p* < 0.0001), except for the 10 μM psilocybin treatment group, which significantly upregulated the IL-6 levels compared to the TNF-α/IFN-γ group (*p* < 0.0001, Figure 9B). Representative protein bands can be seen in Figure 9C.

### 3.5. Anti-Inflammatory Effects of 4-AcO-DMT and Transient Receptor Potential Channel Ligands

**Figure 10:**
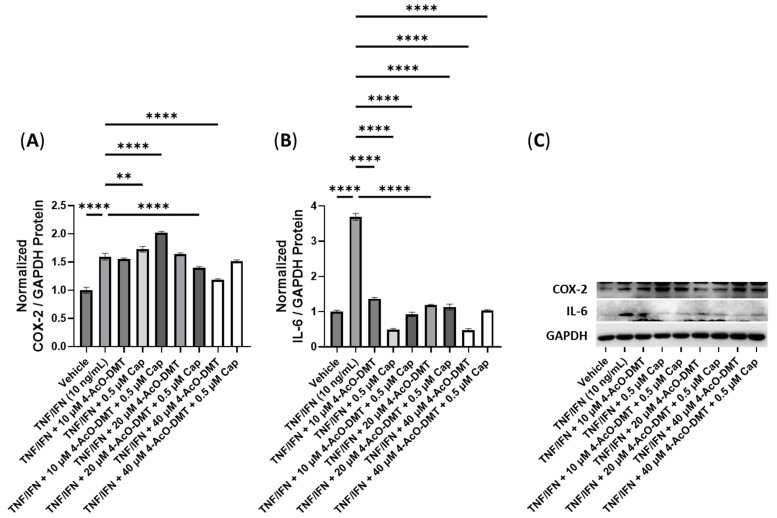
Anti-inflammatory effects of the 4-AcO-DMT and capsaicin cotreatment on (**A**) COX-2 and (**B**) IL-6 protein expression relative to GAPDH. (**C**) Representative Western blots were analyzed for the relative densitometry of the protein expression and normalized to the control. The original Western blots can be found in the Supplementary Materials. Bars represent the mean ± SD. Data were analyzed with a one-way ANOVA test and a Dunnett’s post hoc multiple comparison test compared to the TFN-α/IFN-γ group. Significance is indicated within the figures using the following scale: ** *p* < 0.01, **** *p* < 0.0001. 4-AcO-DMT, 4-acetoxy-N,N-dimethyltryptamine; Cap, capsaicin.

Next, we evaluated the anti-inflammatory effects of combining 4-AcO-DMT with TRP channel ligands. The TNF-α/IFN-γ group exhibited significantly higher levels of COX-2 compared to the control (*p* < 0.0001). However, only the combination of 20 μM of 4-AcO-DMT with 0.5 μM of Cap and 40 μM of 4-AcO-DMT significantly reduced the COX-2 levels compared to the TNF-α/IFN-γ group (*p* < 0.0001, Figure 10A). On the other hand, the relative IL-6 levels were significantly increased in the TNF-α/IFN-γ group compared to the control (*p* < 0.0001), but all treatment groups effectively lowered the IL-6 protein levels compared to the TNF-α/IFN-γ group (*p* < 0.0001, Figure 10B). Representative protein bands can be seen in Figure 10C.

**Figure 11:**
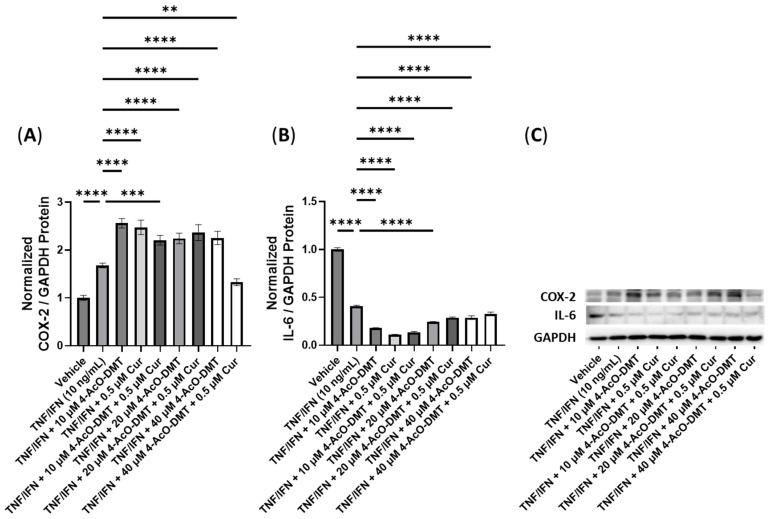
Anti-inflammatory effects of the 4-AcO-DMT and curcumin cotreatment on (**A**) COX-2 and (**B**) IL-6 protein expression relative to GAPDH. (**C**) Representative Western blots were analyzed for the relative densitometry of the protein expression and normalized to the control. The original Western blots can be found in the Supplementary Materials. Bars represent the mean ± SD. Data were analyzed with a one-way ANOVA test and a Dunnett’s post hoc multiple comparison test compared to the TFN-α/IFN-γ group. Significance is indicated within the figures using the following scale: ** *p* < 0.01, *** *p* < 0.001, and **** *p* < 0.0001. 4-AcO-DMT, 4-acetoxy-N,N-dimethyltryptamine; Cur, curcumin.

Subsequently, we examined the effects of combinations of 4-AcO-DMT and Cur on inflammatory response. The COX-2 levels relative to GAPDH were significantly increased in the TNF-α/IFN-γ group compared to the control (*p* < 0.0001). Surprisingly, the COX-2 levels were significantly upregulated in all treatment groups (*p* < 0.001), except for the combination of 40 μM of 4-AcO-DMT with 0.5 μM of Cur, which showed a significant downregulation (*p* < 0.01, Figure 11A). In contrast, the relative protein levels of IL-6 were significantly reduced in all treatment groups (*p* < 0.0001) compared to the TNF-α/IFN-γ group, which exhibited significant downregulation compared to the control (*p* < 0.0001, Figure 11B). Representative protein bands can be seen in Figure 11C.

**Figure 12:**
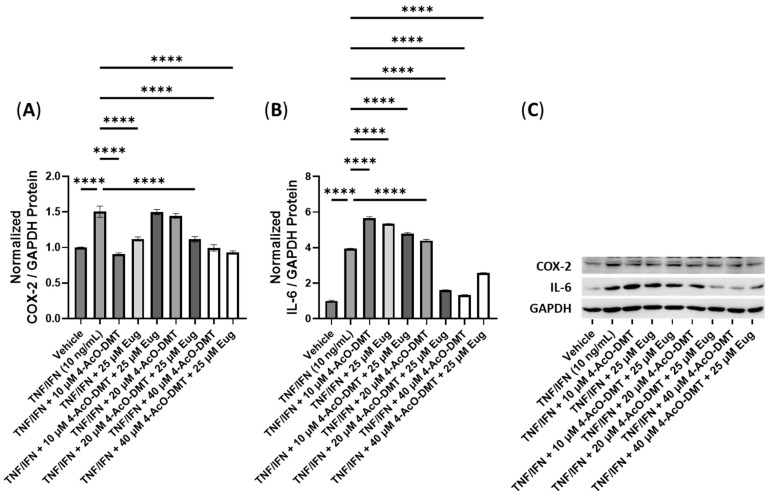
Anti-inflammatory effects of the 4-AcO-DMT and eugenol cotreatment on (**A**) COX-2 and (**B**) IL-6 protein expression relative to GAPDH. (**C**) Representative Western blots were analyzed for the relative densitometry of the protein expression and normalized to the control. The original Western blots can be found in the Supplementary Materials. Bars represent the mean ± SD. Data were analyzed with a one-way ANOVA test and a Dunnett’s post hoc multiple comparison test compared to the TFN-α/IFN-γ group. Significance is indicated within the figures using the following scale: **** *p* < 0.0001. 4-AcO-DMT, 4-acetoxy-N,N-dimethyltryptamine; Eug, eugenol.

Next, we examined the anti-inflammatory properties of combinations of 4-AcO-DMT with eugenol (Figure 12). The relative levels of the COX-2 protein were significantly reduced in the 10 μM of 4-AcO-DMT, 25 μM of Eug, 20 μM of 4-AcO-DMT combined with 25 μM of Eug, 40 μM of 4-AcO-DMT, and 40 μM of 4-AcO-DMT combined with 25 μM of Eug groups compared to the TNF-α/IFN-γ group (*p* < 0.0001, Figure 12A). The TNF-α/IFN-γ group showed significantly higher COX-2 levels compared to the control group (*p* < 0.0001, Figure 12A). Conversely, the relative levels of the IL-6 protein were significantly reduced only in the 20 μM of 4-AcO-DMT combined with 25 μM of Eug, 40 μM of 4-AcO-DMT, and 40 μM of 4-AcO-DMT combined with 25 μM of Eug groups compared to the TNF-α/IFN-γ group (*p* < 0.0001), while the TNF-α/IFN-γ group exhibited significantly higher IL-6 levels than the control group (*p* < 0.0001, Figure 12B). Notably, IL-6 was upregulated in the 10 μM of 4-AcO-DMT, 25 μM of Eug, 10 μM of 4-AcO-DMT combined with 25 μM of Eug, and 20 μM of 4-AcO-DMT group compared to the TNF-α/IFN-γ group (*p* < 0.0001, Figure 12B). Representative protein bands can be seen in Figure 12C.

### 3.6. Anti-Inflammatory Effects of Ketanserin and Transient Receptor Potential Channel Ligands

**Figure 13:**
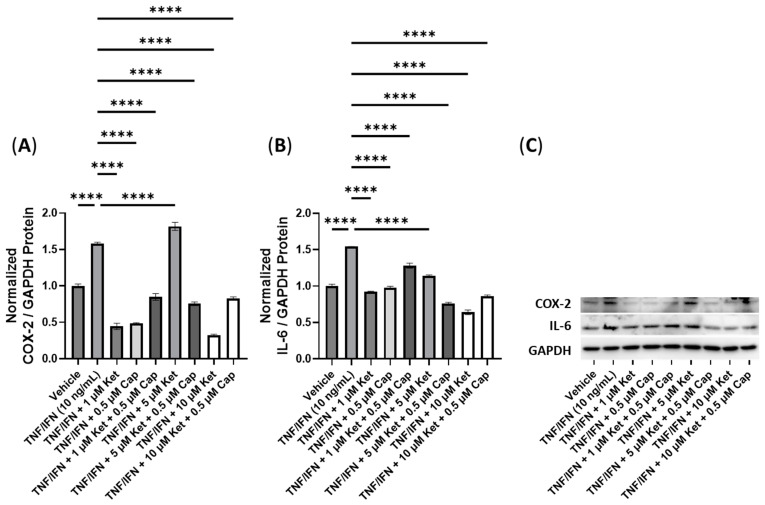
Anti-inflammatory effects of the ketanserin and capsaicin cotreatment on (**A**) COX-2 and (**B**) IL-6 protein expression relative to GAPDH. (**C**) Representative Western blots were analyzed for the relative densitometry of the protein expression and normalized to the control. The original Western blots can be found in the Supplementary Materials. Bars represent the mean ± SD. Data were analyzed with a one-way ANOVA test and a Dunnett’s post hoc multiple comparison test compared to the TFN-α/IFN-γ group. Significance is indicated within the figures using the following scale: **** *p* < 0.0001. Cap, capsaicin; Ket, ketanserin.

Next, we conducted tests to examine the anti-inflammatory effects of ketanserin in combination with TRP channel ligands, starting with capsaicin. Interestingly, the relative protein levels of COX-2 were significantly upregulated by the TNF-α/IFN-γ treatment compared to the control (*p* < 0.0001, Figure 13A). Surprisingly, the 5 μM of Ket treatment significantly upregulated COX-2 protein levels (*p* < 0.0001), while all other treatments significantly reduced COX-2 levels (*p* < 0.0001, Figure 13A). Notably, the 1 μM and 10 μM of Ket treatments appeared to have the most pronounced effect in lowering COX-2 levels (Figure 13A). Additionally, all treatment groups significantly decreased the relative IL-6 protein levels (*p* < 0.0001) compared to the TNF-α/IFN-γ group. IL-6 levels were significantly higher in the TNF-α/IFN-γ group compared to the control (*p* < 0.0001, Figure 13B). For visualization, representative protein bands can be seen in Figure 13C.

**Figure 14:**
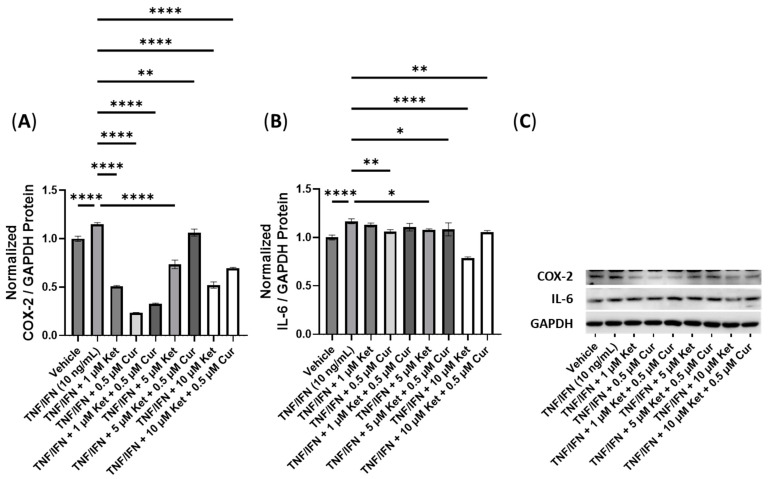
Anti-inflammatory effects of the ketanserin and curcumin cotreatment on (**A**) COX-2 and (**B**) IL-6 protein expression relative to GAPDH. (**C**) Representative Western blots were analyzed for the relative densitometry of the protein expression and normalized to the control. The original Western blots can be found in the Supplementary Materials. Bars represent the mean ± SD. Data were analyzed with a one-way ANOVA test and a Dunnett’s post hoc multiple comparison test compared to the TFN-α/IFN-γ group. Significance is indicated within the figures using the following scale: * *p* < 0.05, ** *p* < 0.01, and **** *p* < 0.0001. Cur, curcumin; Ket, ketanserin.

Moving on, we tested the efficacy of ketanserin in combination with curcumin as anti-inflammatory compounds. The relative COX-2 levels were significantly reduced by 0.5 μM of Cur (*p* < 0.0001), 1 μM of Ket with or without 0.5 μM of Cur (*p* < 0.0001), 5 μM of Ket (*p* < 0.0001), 5 μM of Ket with 0.5 μM of Cur (*p* < 0.01), and 10 μM of Ket with or without 0.5 μM of Cur (*p* < 0.0001) compared to the TNF-α/IFN-γ group, which exhibited upregulation compared to the control (*p* < 0.0001, Figure 14A). Similarly, the relative IL-6 protein levels were significantly increased in the TNF-α/IFN-γ group compared to the control (*p* < 0.0001, Figure 14B). Interestingly, the IL-6 protein levels were significantly reduced by 0.5 μM of Cur (*p* < 0.01), 5 μM of Ket (*p* < 0.05), 5 μM of Ket with 0.5 μM of Cur (*p* < 0.05), 10 μM of Ket (*p* < 0.0001), and 10 μM of Ket with 0.5 μM of Cur (*p* < 0.01) compared to the TNF-α/IFN-γ group. Notably, the 10 μM of Ket group appeared to have the most significant effect in reducing the IL-6 levels (Figure 14B). For visual reference, representative protein bands can be seen in Figure 14C.

**Figure 15:**
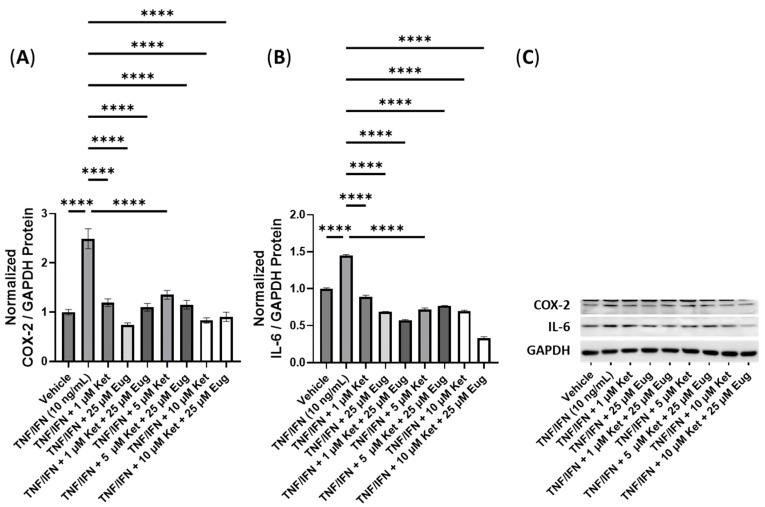
Anti-inflammatory effects of the ketanserin and eugenol cotreatment on (**A**) COX-2 and (**B**) IL-6 protein expression relative to GAPDH. (**C**) Representative Western blots were analyzed for the relative densitometry of the protein expression and normalized to the control. The original Western blots can be found in the Supplementary Materials. Bars represent the mean ± SD. Data were analyzed with a one-way ANOVA test and a Dunnett’s post hoc multiple comparison test compared to the TFN-α/IFN-γ group. Significance is indicated within the figures using the following scale: **** *p* < 0.0001 Eug, eugenol; Ket, ketanserin.

Next, we conducted tests to determine the effectiveness of combining ketanserin with eugenol. All treatments resulted in a significant decrease in COX-2 protein levels compared to the TNF-α/IFN-γ group (*p* < 0.0001). Additionally, the TNF-α/IFN-γ group exhibited a significant upregulation in COX-2 protein levels compared to the control group (*p* < 0.0001, Figure 15A). Similarly, the IL-6 levels were significantly elevated in the TNF-α/IFN-γ group compared to the controls (*p* < 0.0001), but all treatments effectively reduced the IL-6 levels when compared to the TNF-α/IFN-γ group (*p* < 0.0001, Figure 15B). For visualization, representative protein bands can be seen in Figure 15C.

## 4. Discussion

In this study, we aimed to elucidate the anti-inflammatory effects of common 5-HT2A receptor ligands, including psilocybin, 4-AcO-DMT, and ketanserin, as well as TRP channel ligands, including capsaicin, curcumin, and eugenol. Utilizing 24 h TNF-α/IFN-γ exposure, we reliably induced an inflammatory response within HSEIC and tested each compound’s efficacy as an anti-inflammatory agent [25,26]. COX-2, the inducible form of cyclooxygenase, was utilized as the primary measure of the inflammatory response in HSEIC. COX-2 is the key initiator in the inflammatory response by converting arachidonic acid into proinflammatory prostaglandins, which triggers the production of other proinflammatory chemokines, cytokines, and growth factors [27]. In addition, we tested the cytotoxicity of these compounds via MTT assays in a wide array of doses to determine if such treatments may be contraindicated as anti-inflammatory therapeutics.

Psilocybin and 4-AcO-DMT as single treatments in our TNF-α/IFN-γ model did not demonstrate much, if any, cytotoxicity. The only cytotoxicity noted was after 72 h of exposure to 5 and 10 μM of psilocybin (Figure 1A). It is important to note that these effects were not seen at higher doses during the same time period, and the cytotoxic effects at these doses were not seen at any other time points, including time points both before and after 72 h (Figure 1A). Therefore, we believe this finding is a false positive, and psilocybin does not appear to have any cytotoxic effects. This finding may be due to a couple reasons, including a type 1 hypothesis testing error. Importantly, psilocybin and the synthetic form of psilocybin, 4-AcO-DMT, demonstrated consistent and dose-dependent pro-proliferative effects. While psychedelics have been shown to induce pro-proliferative effects in neuronal cultures [28,29] and in vivo in mice [30], to the best of our knowledge, this would be the first study to demonstrate this effect on intestinal or epithelial cells for psilocybin or 4-AcO-DMT.

In contrast, ketanserin did demonstrate both short-term and long-term toxicities at doses of 40 μM and 5 μM or higher, respectively. In addition, ketanserin did not prove to be very effective in lowering the inflammatory response, as measured by COX-2 protein levels. The optimal dose of 1 μM resulted in a fold change of −1.7× in COX-2 levels (Table 1), while higher doses significantly increased COX-2 levels (Figure 6C). Since previous studies have shown selective agonists for other serotonin receptors, including 5-HT2B and 5-HT2C, which did not induce anti-inflammatory effects, the reduction in COX-2 levels seen here can be assumed to be induced through 5-HT2A agonism and signaling [5]. While both Gα_q/11_ and Gα_i/o_ are commonly coupled to 5-HT2A, psilocybin can induce biased signaling, specifically through β-arrestin-2 [31], to negatively regulate NF-κB [32,33]. Potentially, psilocybin and 4-AcO-DMT reduced COX-2 levels and inflammatory cytokines through β-arrestin-2-biased signaling, inhibiting NF-κB-induced inflammation, which was synergistically induced in our TNF-α/IFN-γ model [34]. However, further research is required to elucidate the exact mechanism of psilocybin’s anti-inflammatory effects.

It is important to note that, unlike psilocybin and 4-AcO-DMT, ketanserin is a 5-HT2A antagonist. While ketanserin has been shown to inhibit inflammation by reducing IL-6 and ameliorate kidney fibrosis by decreasing TGF-beta and collagen production [5,35], ketanserin demonstrated a moderate ability to reduce COX-2 levels (−1.7×). Our data suggest that selective 5-HT2A agonists (psilocybin) are stronger anti-inflammatory agents than antagonists, specifically ketanserin (Table 1). While the most efficacious dose of 4-AcO-DMT (20 μM) resulted in similar fold changes in COX-2 protein levels (−1.5×), the most efficacious dose of psilocybin (40 μM) resulted in a −3.8× fold change in COX-2 protein levels compared to the TNF-α/IFN-γ treatment (Table 1). Due to the large change in COX-2 levels and no detected toxicity, psilocybin appears to be the most effective single anti-inflammatory therapeutic out of the compounds tested.

Recently, whole mushroom extracts containing psilocybin have been tested and shown to reduce inflammatory responses induced in macrophages. Psilocybin-containing mushrooms were extracted with boiling water and applied to mouse RAW 264.7 macrophages [36] and human U937 macrophages [37]. In mouse macrophages, both water and ethanol extracts significantly reduced LPS-induced prostaglandin and IL-1β protein production [36]. In contrast, psilocybin-containing mushroom extracts significantly lowered COX-2, TNF-α, IL-1β, and IL-6 protein abundance [37]. While both studies did not test psilocybin alone but instead tested an extract that contained psilocybin and multiple compounds, psilocybin was likely the compound responsible for these effects due to the presence of 5-HT2A receptors in macrophages. These studies are in line with our data suggesting psilocybin can strongly inhibit inflammation (Figure 6). While similar compounds with an affinity for serotonin receptors can have anti-inflammatory effects, like 4-AcO-DMT and ketanserin, we found psilocybin had a far superior anti-inflammatory effect in the intestinal epithelium (Table 1).

In contrast, eugenol demonstrated both short- and long-term cytotoxicity that might contraindicate its use as an anti-inflammatory therapeutic. Eugenol demonstrated cytotoxicity at doses of 10 μM or higher, although its most efficacious dose was 50 μM; at a dose of 50 μM of eugenol, COX-2 levels decreased by 3.8× (Table 1), demonstrating strong anti-inflammatory effects.

Previous studies have suggested that eugenol has considerable anti-inflammatory activity. In porcine intestinal epithelial cells, a pretreatment with 100 μM of eugenol could significantly suppress the LPS-induced *IL-8* and *TNF-α* transcript levels, restoring tight junction proteins and transporters while improving the barrier integrity [17]. In addition, eugenol has shown strong anti-inflammatory properties in a dextran sulfate sodium-induced colitis mouse model. Eugenol at a dose of 20 mg/kg, consumed orally for 17 days, reduced the pathological scores; prevented oxidative stress; lowered the IL-6, IL-12, IL-21, and IL-23 proinflammatory cytokines; and attenuated NF-κB signaling by decreasing p65 phosphorylation [38]. While many have hypothesized that the effects of eugenol may be due to altering the gut microbiome, eugenol’s effects appear to be independent of alterations to the gut microbiome [38]. In line with the gut microbiome-independent effects, our results show eugenol can ameliorate inflammatory responses induced by TNF-α/IFN-γ by directly acting on the intestinal epithelium. In addition, eugenol is known to interact with multiple TRP channels to induce CaMKK2 signaling [39], which suppresses chemokine production in myeloid subsets [40].

Curcumin demonstrated short-term cytotoxicity at doses of 5 μM of higher and long-term cytotoxicity at doses of 1 μM of higher. However, at the most effective dose of 0.5 μM, curcumin can act as an anti-inflammatory agent and decrease COX-2 levels by 3.8× fold without causing cytotoxic effects (Table 1). Our results match previous data showing curcumin has potent anti-inflammatory effects [14].

Curcumin has been shown to prevent inflammatory responses by binding to toll-like receptors (TLR) and regulating downstream signaling [41]. This includes NF-κB, mitogen-activated protein kinases, and activator protein 1. Curcumin can downregulate NF-κB by acting on peroxisome proliferator-activated receptor gamma and, as a result, prevent the assembly and activation of the NOD-like receptor pyrin domain-containing 3 (NLRP3) inflammasome. Curcumin can also act on TRPV1 channels. TRPV1 channels are important immunomodulators in IBD and are found on macrophages, T cells, and leukocytes [42]. The oral administration of curcumin in dextran sodium sulphate (DSS)-treated mice has been shown to ameliorate visceral hyperalgesia by inhibiting TRPV1 phosphorylation; however, it is currently unknown if curcumin inhibits inflammation through TRPV1 signaling [42].

In contrast, capsaicin did not demonstrate significant cytotoxicity in either the short- or long-term doses tested (Table 1); however, capsaicin did not greatly decrease COX-2 levels at the most efficacious dose. At a dose of 0.5 μM, capsaicin resulted in a COX-2 fold change of −1.6×. Despite these findings, capsaicin has demonstrated strong anti-inflammatory effects in other in vitro studies [43,44].

Although capsaicin had minimal effects on altering the inflammatory response seen in our TNF-α/IFN-γ model of inflammation in HSEIC, capsaicin can decrease the expression of LPS/TLR4-induced proinflammatory cytokines, including IL-8 and TNF-α, in porcine intestinal epithelial cells [44] and reduce TLR5/NF-κB-induced TNF-α in human colon HT-29 cells [43]. The differences between our results and previously published data that demonstrate capsaicin prevents inflammation could be due to the type of model utilized. We used TNF-α/IFN-γ, while other studies utilized LPS or *Clostridioides difficile* toxins, which operate through TLRs [43,44]. In other models that induce TLR/NF-κB signaling, capsaicin reduced TLR/NF-κB signaling and the resulting inflammation, while, in our TNF-α/IFN-γ model, which does not induce NF-κB through TLRs [45], capsaicin had minimal effects. In addition, capsaicin can activate TRPV1 channels and has been shown to decrease DSS-induced colitis in rats when delivered subcutaneously [46]. In contrast, orally consumed capsaicin appears to be able to induce or worsen chronic colitis in some models [47]. This may be due to capsaicin inducing dysbiosis—specifically, reducing Firmicutes and increasing Bacteroides—resulting in intestinal permeability and the translocation of gut pathogen molecules to exacerbate inflammation [48].

Next, we tested if combinations of a serotonin receptor ligands combined with a TRP channel ligands could demonstrate synergistic effects to reduce COX-2 levels without causing cytotoxic effects. To do this, we tested a range of doses that would likely show beneficial results based on Figure 1, Figure 2 and Figure 6. In addition, we tested the effects on the IL-6 levels.

IL-6 is pleiotropic cytokine that aids in maintaining musical integrity and defends against invasive pathogens, while also regulating intestinal motility and secretion by acting on smooth muscle cells and secretory cells in physiological settings [49]. Normally, IL-6 can induce acute inflammation and reduce bacterial colonization; however, in pathological conditions, excessive and prolonged IL-6 production by the intestinal epithelium increases the immune cell population and causes chronic inflammation, leading to IBD [50]. By reducing the IL-6 levels, the disease progression and severity of IBD can be reduced [51].

Psilocybin combined with capsaicin did not appear to show synergistic effects on the COX-2 levels compared to psilocybin alone at the most efficacious dose (Table 1). In contrast, both psilocybin with curcumin and psilocybin with eugenol demonstrated synergistic effects. The most effective dose was 40 μM of psilocybin and 0.5 μM of curcumin and resulted in a COX-2 fold change of −28× and an IL-6 fold change of −1.6× (Table 1). While no cytotoxic effects were seen at this dose, lower doses of psilocybin and curcumin did induce cytotoxic effects. In contrast, psilocybin and eugenol demonstrated the best ability to lower IL-6 levels out of all psilocybin combinations. The combination of 40 μM of psilocybin and 25 μM of eugenol resulted in a fold change of −10× for IL-6 and −19× for COX-2 (Table 1). Due to these synergistic effects and lack of cytotoxicity, combinations of psilocybin and eugenol show promise as future anti-inflammatory therapeutics.

Interestingly, the oral consumption of psilocybin and eugenol has previously been shown to be effective in vivo in lowering brain inflammation in mice. In ratios of 1:10, 1:20, and 1:50, psilocybin and eugenol were tested on their efficacy to prevent LPS-induced brain inflammation. The ratio of 1:50 with 0.88 mg/kg of psilocybin demonstrated the best results in reducing COX-2, TNF-α, Il-1β, and IL-6. In this study, our results demonstrated similar synergistic effects to lower COX-2 and IL-6 levels in epithelial intestinal cells (Table 1).

Psilocybin and eugenol act on different receptors and are believed to have different signaling transductions, which could explain their synergistic actions to reduce proinflammatory cytokine production. Psilocybin acts primarily through the 5-HT2A receptor, likely acting through Gα_q/11_, Gα_i/o_, and/or β-arrestin-2, whereas, eugenol can interact with TRPV1 to induce CaMKK2 signaling [39], thereby suppressing chemokine production in myeloid subsets [40]. Due to these differing anti-inflammatory mechanisms, it is not surprising that eugenol and psilocybin can have potent and synergistic anti-inflammatory effects.

In contrast, neither curcumin nor capsaicin had synergistic effects. Curcumin has known anti-inflammatory properties, reducing NF-κB activation and NLRP3 activation, and can act on TRPV3 channels as well [41]. As such, it is surprising that curcumin did not have a synergistic effect with psilocybin. It is possible that most of the effects induced by curcumin are mediated through NF-κB. Since psilocybin can induce β-arrestin-2 signaling, which inhibits NF-κB activation [31,32], the effects of curcumin may be moot, as NF-κB is already inhibited. Furthermore, capsaicin is known to similarly mediate its effects through TLR/NF-κB signaling and, as such, could not have a synergistic effect [44].

Next, we tested the anti-inflammatory effects of 4-AcO-DMT combined with capsaicin, curcumin, and eugenol. While none of the combinations resulted in short-term cytotoxicity, 10 μM of 4-AcO-DMT and ≥0.5 μM of curcumin did cause long-term cytotoxicity (Table 1). The most effective doses were found to be 20 μM of 4-AcO-DMT with 0.5 μM of capsaicin, 40 μM of 4-AcO-DMT with 0.5 μM of curcumin, and 40 μM of 4-AcO-DMT with 25 μM of eugenol, which had fold changes of −1.1× and −3.3×, −1.3× and −1.3×, and −1.6× and −1.5× for COX-2 and IL-6 protein levels, respectively. All three combinations did not appear to have synergistic effects on their ability to lower COX-2 levels; however, 4-AcO-DMT with capsaicin appeared to be more effective in lowering the IL-6 levels than all other combinations, except for 40 μM of psilocybin and 25 μM of eugenol (Table 1). The differences in synergy between psilocybin and 4-AcO-DMT can likely be contributed to ligand specificity at the 5-HT2A receptor resulting in different downstream signaling [52,53].

Lastly, the ketanserin combinations did not show any short-term cytotoxicity in vitro; however, both 10 μM of ketanserin with 0.5 μM of capsaicin and ≥1 μM of ketanserin with 25 μM of eugenol were cytotoxic after longer exposures (Table 1). In addition, all three combinations did not appear to have any synergistic effects on reducing COX-2 levels, while some combinations did decrease the fold changes in the IL-6 levels. The combination of 10 μM of ketanserin with 25 μM of eugenol demonstrated the largest decrease in the IL-6 levels out of all ketanserin combinations with a fold change of −3.0×; however, this dose demonstrated cytotoxic effects (Table 1). The combination of 5 μM of ketanserin with 0.5 μM of capsaicin had a fold change of −2.0× for IL-6 levels and did not show any cytotoxic effects; however, other serotonin and TRP channel ligands demonstrated superior results (Table 1).

While synergy between ketanserin and eugenol or capsaicin is intriguing, their clinical use is contraindicated due to the known adverse effects of ketanserin. Nevertheless, the anti-inflammatory effects seen through 5-HT2A receptor antagonism are known to be mediated through both MEK/ERK to reduce nitrosative stress [54] and have been shown to inhibit IL-6 production as well [35,54,55]. As MEK/ERK both regulate IL-6 production through Stat3 signaling [56], this is likely how ketanserin can further reduce IL-6 expression when ketanserin is combined with TRP ligands that are not known to interact with the MEK/ERK/STAT3/IL-6 pathway. In contrast, serotonin is known to affect prostaglandin production [57] and increase COX-2 [58]; however, the effects of 5-HT2A antagonism on COX-2 levels are not currently known other than ameliorating the agonist-induced COX-2 levels [59].

Out of all the treatments, 40 μM of psilocybin combined with 25 μM of eugenol showed the greatest promise to reduce the inflammatory response without causing cytotoxic effects. These anti-inflammatory effects appeared to be synergistic, as there was a higher magnitude of change compared to a single treatment of either compound for all tested concentrations. These effects may be mediated by both serotonin receptors, 5-HT2A and 5-HT2B, as well as TRPV1 and TRPM8; however, future studies should examine the mechanism of how these synergistic anti-inflammatory effects are propagated. Not only is this the first study to demonstrate the synergistic effects of serotonin ligands and TRP channel ligands as anti-inflammatories in the intestines, but to our knowledge, this is the first study to demonstrate that either psilocybin or 4-AcO-DMT have anti-inflammatory effects in the intestines and could be utilized to assist in inflammatory bowel diseases.

In recent years, there has been growing interest in exploring the potential of serotonin receptor ligands as anti-inflammatory agents. Within the intestines, approximately 95% of all 5-HT produced within the body is synthesized by enterochromaffin cells, which are regulated by the gut microbiota [60]. 5-HT found within the gut is known to play multiple roles, modulating the gut motility, secretion, inflammation, and metabolic homeostasis [60] and regulating the intestinal permeability [61], as well as many other functions [60]. The activation of certain serotonin receptors [11,62] has been shown to modulate immune responses and improve barrier function in the intestinal epithelium by acting directly on immune cells [63]. By increasing serotonergic activation within the intestines, researchers have been able to reduce inflammation within the gut and prevent the development of IBD [64]. Interestingly, the 5-HT1, 5-HT3, and 5-HT4 receptors have been studied and are known to alter intestinal inflammation; however, the effects of 5-HT2 receptor activation on intestinal inflammation have been poorly studied.

One study examined the effect of the selective 5-HT2A ligand (R)-DOI on intestinal inflammation in vivo. They found (R)-DOI was able to reduce the TNF-induced *mcp-1*, *il-6*, and *il-1beta* gene expression levels in the intestines; however, the protein levels were not measured [65]. More recently, 2,4,6-trinitrobenzene sulfonic acid-induced colitis was shown to be regulated primarily by 5-HT2A, instead of the 5-HT1A, 5-HT3, 5-HT4, and 5-HT7 receptors. In addition, knockout of the *Htr2b* gene, which encodes the 5-HT2B receptor, induces colitis and promotes colitis-associated cancer [66]. Despite these findings indicating the importance of 5-HT2 receptors in regulating intestinal inflammation, psilocybin, 4-AcO-DMT, and other 5-HT2 receptor ligands have not been studied.

While the effects of (R)-DOI are commonly generalized to psilocybin, the 5-HT2A receptor demonstrates marked biased agonism [24] due to different receptor binding pockets [52] and downstream signaling [67]. Instead of binding solely to the orthosteric binding pocket, psilocybin can also bind to the adjacent region known as the extended binding pocket [52]. This alternative binding mode leads to biased activation of a protein called β-arrestin2, which is associated with the recruitment of different signaling pathways [52]. Due to biased signaling and different affinities to the 5-HT2 receptors, the effects of 5-HT2 ligands should each be tested and their effects compared. The few studies that have tested psilocybin as a potential anti-inflammatory therapeutic in immune cells have shown potent anti-inflammatory effects [5]. In our study, we demonstrate the differences between 5-HT2A receptor ligands, including similar agonists such as psilocybin and 4-AcO-DMT, as well as compared to ketanserin, an antagonist.

Similarly, TRP channels have emerged as potential therapeutic targets for IBD. TRP channels are ion channels that are involved in sensing and responding to diverse stimuli, including inflammation [68]. Ligands of specific TRP channels, such as TRPV1, have shown anti-inflammatory effects in preclinical studies by inhibiting the release of proinflammatory mediators and promoting epithelial barrier integrity [69]. In addition, TRPM8 activation has been shown to attenuate inflammatory responses in mouse models of colitis [70], while *Trpm8* mouse knockouts are hypersensitive to chemical-induced colitis [71]. Due to these studies showing TRP channel activity in animal models is effective in reducing inflammation in the intestinal epithelium, TRP channel ligands hold promise as therapeutics for humans to prevent or attenuate IBD and the associated inflammation. Importantly, we demonstrate within this study that specific TRP channel ligands can synergistically reduce inflammatory responses within HSEIC.

While the use of serotonin receptor ligands and TRP channel ligands as anti-inflammatories in the epithelium of the intestines for IBD prevention is still in the experimental stages, the early findings are promising, but there are many limitations to our study and the use of 5-HT2A and TRP channel ligands for the treatment of IBD.

In this study, we tested the anti-inflammatory response of HSEIC in response to the TNF-α/IFN-γ treatment. While COX-2 and IL-6 are important mediators of IBD, inflammation and IBD involve numerous cytokines and interconnecting signaling pathways between HSEIC and immune cells [72]. Future studies should investigate the efficacy of these synergistic drug combinations within animal models to further validate the anti-inflammatory effects seen within this study. Furthermore, there are numerous 5-HT2A receptor and TRP channel ligands. We studied a specific and limited subset of ligands for these receptors that cannot be generalized to other ligands due to differences in their binding selectivity and affinity.

In addition, there is a legitimate concern for utilizing psychedelics as anti-inflammatory agents due to the hallucinogenic effects of psilocybin and 4-AcO-DMT. However, microdosing has become an increasingly common phenomenon due to the beneficial effects of microdosing without any typical hallucinogenic effects and minimal side effects [73]. Utilizing doses of psilocybin that can induce anti-inflammatory effects but are below the required dose to act as hallucinogens could circumvent these issues. As such, we tested combinations of 5-HT2A receptor ligands with TRP channel ligands. Due to the synergistic effects seen between these two classes of ligands, the required dose of psilocybin can potentially be reduced to sub-hallucinogenic while increasing the therapeutic window and still induce similar anti-inflammatory effects compared to psilocybin alone. While infrequent psilocybin use has been shown to be relatively safe when using a controlled set and in a controlled setting, even at hallucinogenic doses [74], the long-term effects of frequent sub-hallucinogenic doses, known as microdosing, is not known. Furthermore, multiple scientific studies and authorities recognize the urgent need to study the safety of the reoccurring therapeutic modulation of serotonin signaling via microdosing with psilocybin [75,76,77]. In contrast, the benefits are consistently shown with large sample sizes; however, the current studies have a gender bias while lacking heterogeneity in the dosing schedules and concurrent drug use [76].

Since ketanserin is a prescribed drug, used to manage preeclampsia and to treat hypertension, as well as chronic ulcers in leprosy and diabetic patients [78], there are known adverse effects in humans. The adverse effects include prolonging the cardiac QT interval, inducing long QT syndrome and potentially inducing cardiac arrythmias [78]. In addition, ketanserin can induce drowsiness, fatigue, headaches, sleep disturbances, lack of concentration, dyspepsia [79], and orthostatic hypotension [80]. While ketanserin does demonstrate anti-inflammatory properties and has clinical indications, its known adverse reactions would prevent the use of ketanserin as an anti-inflammatory drug.

Furthermore, orally consumed capsaicin would likely not be used as a therapeutic treatment of IBD due to its known effects on the microbiota. Capsaicin can reduce Firmicutes and increase Bacteroides, resulting in intestinal permeability and endotoxemia, which would exacerbate inflammation [48].

While this study establishes synergistic effects between psilocybin and eugenol without any observed cytotoxic effects, further research is required. Future studies should determine the efficacy of psilocybin and eugenol by studying their effects beyond cellular models of inflammatory intestinal diseases. Before human consumption, clinical trials should be performed, and their safety profiles and potential as therapeutic interventions for IBD still need to be assessed.

## 5. Conclusions

Both 5-HT2A ligands and TRP channel ligands demonstrate promise in reducing the inflammatory response within the intestinal epithelium. As single treatments, psilocybin, 4-AcO-DMT, and curcumin can reduce COX-2 levels substantially. While eugenol can lower COX-2 levels as well, eugenol demonstrates cytotoxicity at the relevant doses. In contrast, combinations of psilocybin and eugenol do not demonstrate any cytotoxic effects and appear to have synergistic effects to substantially lower COX-2 and IL-6 protein levels. Further preclinical and clinical research should test the anti-inflammatory efficacy of psilocybin combined with eugenol in vivo.

## Figures and Tables

**Table 1:** Summary of the cytotoxicity, most effective doses, and fold changes of COX-2 and IL-6 after single and combined doses of psilocybin, 4-AcO-DMT, ketanserin, capsaicin, curcumin, and eugenol in HSEIC.

Compounds	Short-Term Cytotoxicity	Long-Term Cytotoxicity	Most Effective Dose	Fold Change of COX-2	Fold Change of IL-6
Psi	N/A	N/A	40 μM	−3.8×	N/A
4-AcO-DMT	N/A	N/A	20 μM	−1.5×	N/A
Ket	≥40 μM	≥5 μM	1 μM	−1.7×	N/A
Cap	≥50 μM	≥0.5 μM	0.5 μM	−1.6×	N/A
Cur	≥5 μM	≥1 μM	0.5 μM	−3.8×	N/A
Eug	≥10 μM	≥10 μM	50 μM	−3.8×	N/A
Psi + Cap	N/A	10 μM Psi, ≥0.5 μM Cap	20 μM Psi, 0.5 μM Cap	−3.8×	−1.7×
Psi + Cur	10 μM Psi & 1 μM Cur	10/20 μM Psi, ≥0.5 μM Cur	40 μM Psi, 0.5 μM Cur	−28×	−1.6×
Psi + Eug	N/A	N/A	40 μM Psi, 25 μM Eug	−19×	−10×
4-AcO-DMT + Cap	N/A	N/A	20 μM 4-AcO-DMT, 0.5 μM Cap	−1.1×	−3.3×
4-AcO-DMT + Cur	N/A	10 μM 4-AcO-DMT, ≥0.5 μM Cur	40 μM 4-AcO-DMT, 0.5 μM Cur	−1.3×	−1.3×
4-AcO-DMT + Eug	N/A	N/A	40 μM 4-AcO-DMT, 25 μM Eug	−1.6×	−1.5×
Ket + Cap	N/A	N/A	5 μM Ket, 0.5 μM Cap	−2.1×	−2.0×
Ket + Cur	N/A	10 μM Ket, ≥0.5 μM Cur	1 μM Ket, 0.5 μM Cur	−3.5×	−1.1×
Ket + Eug	N/A	≥1 μM Ket, 25 μM Eug	10 μM Ket, 25 μM Eug	−2.8×	−3.0×

4-AcO-DMT, 4-acetoxy-N,N-dimethyltryptamine; Cap, capsaicin; Cur, curcumin; Eug, eugenol; Ket, ketanserin; Psi, psilocybin.

## Data Availability

The original blots can be obtained in the supplementary figures.

## Supplementary Materials

The following supporting information can be downloaded at https://www.mdpi.com/article/10.3390/cimb45080427/s1: Figure S1. The original Western blots of HSEIC proteins showing COX-2 at the molecular weight of 72 kDa. Red arrow indicates the bands shown in Figure 6A. Figure S2. The original Western blots of HSEIC proteins showing GAPDH at the molecular weight of 36 kDa. Red arrow indicates the bands shown in Figure 6A. Figure S3. The original Western blots of HSEIC proteins showing COX-2 at the molecular weight of 72 kDa. Red arrow indicates the bands shown in Figure 6B. This blot membrane has been cut into pieces. Each membrane received different exposure times, and the images were taken separately. Figure S4. The original Western blots of HSEIC proteins showing GAPDH at the molecular weight of 36 kDa. Red arrow indicates the bands shown in Figure 6B. This blot membrane has been cut into pieces. Each membrane received different exposure times, and the images were taken separately. Figure S5. The original Western blots of HSEIC proteins showing COX-2 at the molecular weight of 72 kDa. Red arrow indicates the bands shown in Figure 6C. Figure S6. The original Western blots of HSEIC proteins showing GAPDH at the molecular weight of 36 kDa. Red arrow indicates the bands shown in Figure 6C. Figure S7. The original Western blots of HSEIC proteins showing COX-2 at the molecular weight of 72 kDa. Red arrow indicates the bands shown in Figure 6D. Figure S8. The original Western blots of HSEIC proteins showing GAPDH at the molecular weight of 36 kDa. Red arrow indicates the bands shown in Figure 6D. Figure S9. Original Western blots of HSEIC proteins showing COX-2 at molecular weight of 72 kDa. Red arrow indicates bands shown in Figure 6E. This blot membrane has been cut into pieces. Each membrane received different exposure times, and the images were taken separately. Figure S10. The original Western blots of HSEIC proteins showing GAPDH at the molecular weight of 36 kDa. Red arrow indicates the bands shown in Figure 6E. This blot membrane has been cut into pieces. Each membrane received different exposure times, and the images were taken separately. Figure S11. The original Western blots of HSEIC proteins showing COX-2 at the molecular weight of 72 kDa. Red arrow indicates the bands shown in Figure 6F. This blot membrane has been cut into pieces. Each membrane received different exposure times, and the images were taken separately. Figure S12. The original Western blots of HSEIC proteins showing GAPDH at the molecular weight of 36 kDa. Red arrow indicates the bands shown in Figure 6F. This blot membrane has been cut into pieces. Each membrane received different exposure times, and the images were taken separately. Figure S13. The original Western blots of HSEIC proteins showing COX-2 at the molecular weight of 72 kDa. Red arrow indicates the bands shown in Figure 7C. This blot membrane has been cut into pieces. Each membrane received different exposure times, and the images were taken separately. Figure S14. The original Western blots of HSEIC proteins showing IL-6 at the molecular weight of 21 kDa. Red arrow indicates the bands shown in Figure 7C. This blot membrane has been cut into pieces. Each membrane received different exposure times, and the images were taken separately. Figure S15. The original Western blots of HSEIC proteins showing GAPDH at the molecular weight of 36 kDa. Red arrow indicates the bands shown in Figure 7C. This blot membrane has been cut into pieces. Each membrane received different exposure times, and the images were taken separately. Figure S16. The original Western blots of HSEIC proteins showing COX-2 at the molecular weight of 72 kDa. Red arrow indicates the bands shown in Figure 8C. This blot membrane has been cut into pieces. Each membrane received different exposure times, and the images were taken separately. Figure S17. The original Western blots of HSEIC proteins showing IL-6 at the molecular weight of 21 kDa. Red arrow indicates the bands shown in Figure 8C. This blot membrane has been cut into pieces. Each membrane received different exposure times, and the images were taken separately. Figure S18. The original Western blots of HSEIC proteins showing GAPDH at the molecular weight of 36 kDa. Red arrow indicates the bands shown in Figure 8C. This blot membrane has been cut into pieces. Each membrane received different exposure times, and the images were taken separately. Figure S19. The original Western blots of HSEIC proteins showing COX-2 at the molecular weight of 72 kDa. Red arrow indicates the bands shown in Figure 9C. This blot membrane has been cut into pieces. Each membrane received different exposure times, and the images were taken separately. Figure S20. The original Western blots of HSEIC proteins showing IL-6 at the molecular weight of 21 kDa. Red arrow indicates the bands shown in Figure 9C. This blot membrane has been cut into pieces. Each membrane received different exposure times, and the images were taken separately. Figure S21. The original Western blots of HSEIC proteins showing GAPDH at the molecular weight of 36 kDa. Red arrow indicates the bands shown in Figure 9C. This blot membrane has been cut into pieces. Each membrane received different exposure times, and the images were taken separately. Figure S22. The original Western blots of HSEIC proteins showing COX-2 at the molecular weight of 72 kDa. Red arrow indicates the bands shown in Figure 10C. This blot membrane has been cut into pieces. Each membrane received different exposure times, and the images were taken separately. Figure S23. The original Western blots of HSEIC proteins showing IL-6 at the molecular weight of 21 kDa. Red arrow indicates the bands shown in Figure 10C. This blot membrane has been cut into pieces. Each membrane received different exposure times, and the images were taken separately. Figure S24. The original Western blots of HSEIC proteins showing GAPDH at the molecular weight of 36 kDa. Red arrow indicates the bands shown in Figure 10C. This blot membrane has been cut into pieces. Each membrane received different exposure times, and the images were taken separately. Figure S25. The original Western blots of HSEIC proteins showing COX-2 at the molecular weight of 72 kDa. Red arrow indicates the bands shown in Figure 11C. This blot membrane has been cut into pieces. Each membrane received different exposure times, and the images were taken separately. Figure S26. The original Western blots of HSEIC proteins showing IL-6 at the molecular weight of 21 kDa. Red arrow indicates the bands shown in Figure 11C. This blot membrane has been cut into pieces. Each membrane received different exposure times, and the images were taken separately. Figure S27. The original Western blots of HSEIC proteins showing GAPDH at the molecular weight of 36 kDa. Red arrow indicates the bands shown in Figure 11C. This blot membrane has been cut into pieces. Each membrane received different exposure times, and the images were taken separately. Figure S28. The original Western blots of HSEIC proteins showing COX-2 at the molecular weight of 72 kDa. Red arrow indicates the bands shown in Figure 12C. This blot membrane has been cut into pieces. Each membrane received different exposure times, and the images were taken separately. Figure S29. The original Western blots of HSEIC proteins showing IL-6 at the molecular weight of 21 kDa. Red arrow indicates the bands shown in Figure 12C. This blot membrane has been cut into pieces. Each membrane received different exposure times, and the images were taken separately. Figure S30. The original Western blots of HSEIC proteins showing GAPDH at the molecular weight of 36 kDa. Red arrow indicates the bands shown in Figure 12C. This blot membrane has been cut into pieces. Each membrane received different exposure times, and the images were taken separately. Figure S31. The original Western blots of HSEIC proteins showing COX-2 at the molecular weight of 72 kDa. Red arrow indicates the bands shown in Figure 13C. This blot membrane has been cut into pieces. Each membrane received different exposure times, and the images were taken separately. Figure S32. The original Western blots of HSEIC proteins showing IL-6 at the molecular weight of 21 kDa. Red arrow indicates the bands shown in Figure 13C. This blot membrane has been cut into pieces. Each membrane received different exposure times, and the images were taken separately. Figure S33. The original Western blots of HSEIC proteins showing GAPDH at the molecular weight of 36 kDa. Red arrow indicates the bands shown in Figure 13C. This blot membrane has been cut into pieces. Each membrane received different exposure times, and the images were taken separately. Figure S34. The original Western blots of HSEIC proteins showing COX-2 at the molecular weight of 72 kDa. Red arrow indicates the bands shown in Figure 14C. This blot membrane has been cut into pieces. Each membrane received different exposure times, and the images were taken separately. Figure S35. The original Western blots of HSEIC proteins showing IL-6 at the molecular weight of 21 kDa. Red arrow indicates the bands shown in Figure 14C. This blot membrane has been cut into pieces. Each membrane received different exposure times, and the images were taken separately. Figure S36. The original Western blots of HSEIC proteins showing GAPDH at the molecular weight of 36 kDa. Red arrow indicates the bands shown in Figure 14C. This blot membrane has been cut into pieces. Each membrane received different exposure times, and the images were taken separately. Figure S37. The original Western blots of HSEIC proteins showing COX-2 at the molecular weight of 72 kDa. Red arrow indicates the bands shown in Figure 15C. This blot membrane has been cut into pieces. Each membrane received different exposure times, and the images were taken separately. Figure S38. The original Western blots of HSEIC proteins showing IL-6 at the molecular weight of 21 kDa. Red arrow indicates the bands shown in Figure 15C. This blot membrane has been cut into pieces. Each membrane received different exposure times, and the images were taken separately. Figure S39. The original Western blots of HSEIC proteins showing GAPDH at the molecular weight of 36 kDa. Red arrow indicates the bands shown in Figure 15C. This blot membrane has been cut into pieces. Each membrane received different exposure times, and the images were taken separately.
