# Supplementary material for: Anti-Inflammatory Effects of Serotonin Receptor and Transient Receptor Potential Channel Ligands in Human Small Intestinal Epithelial Cells
Source: Curr Issues Mol Biol. 2023 Aug 15;45(8):6743–74. doi: 10.3390/cimb45080427 (PMC10453699; doi:10.3390/cimb45080427)
Supplement: Supplementary file 1 [file cimb-45-00427-s001.zip › cimb-2534526-supplementary.pdf]

Supplementary Materials

COX-2 (72 kDa) in Figure 6A

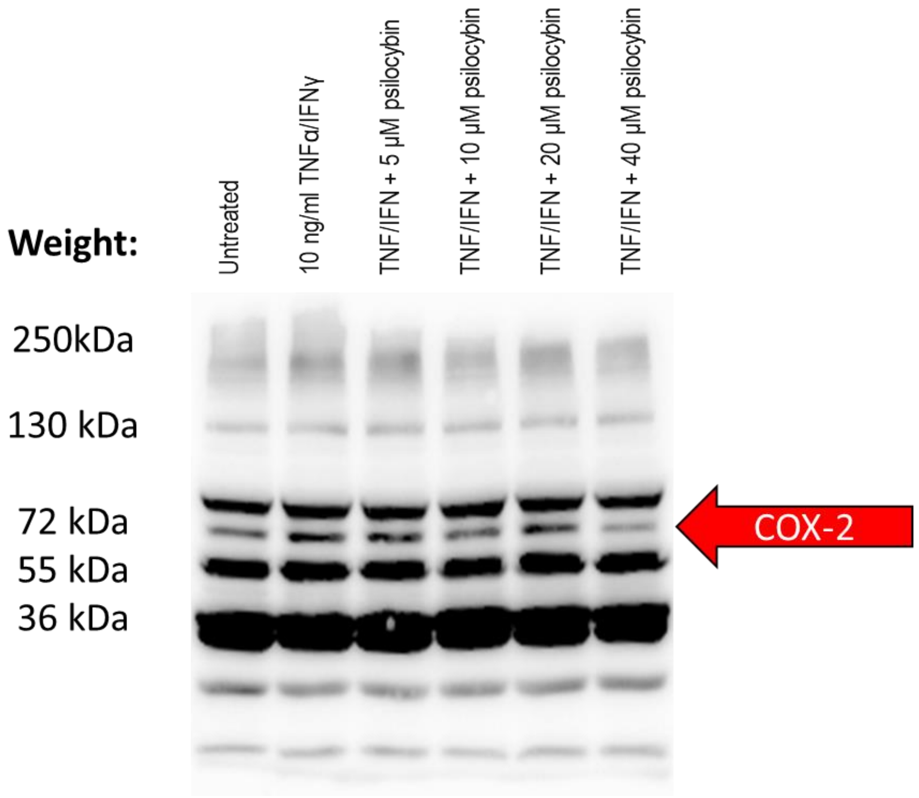

Figure S1. Original Western blots of HSEIC proteins showing COX-2 at molecular weight of 72 kDa. Red arrow indicates bands shown in Figure 6A.

GAPDH (36 kDa) in Figure 6A

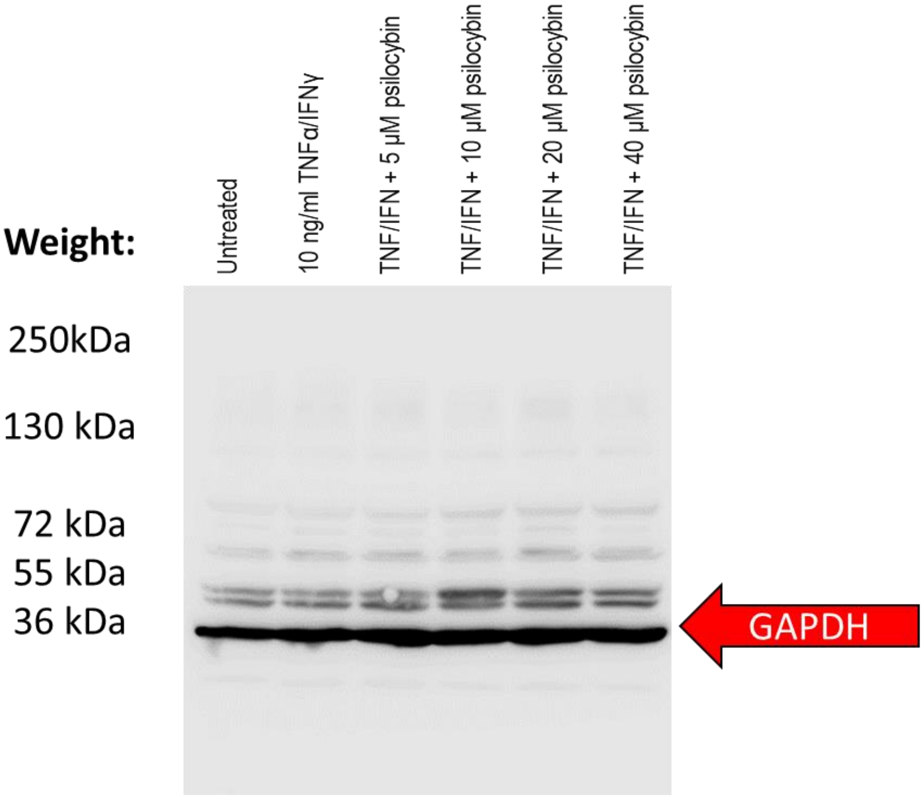

Figure S2. Original Western blots of HSEIC proteins showing GAPDH at molecular weight of 36 kDa. Red arrow indicates bands shown in Figure 6A.

COX-2 (72 kDa) in Figure 6B

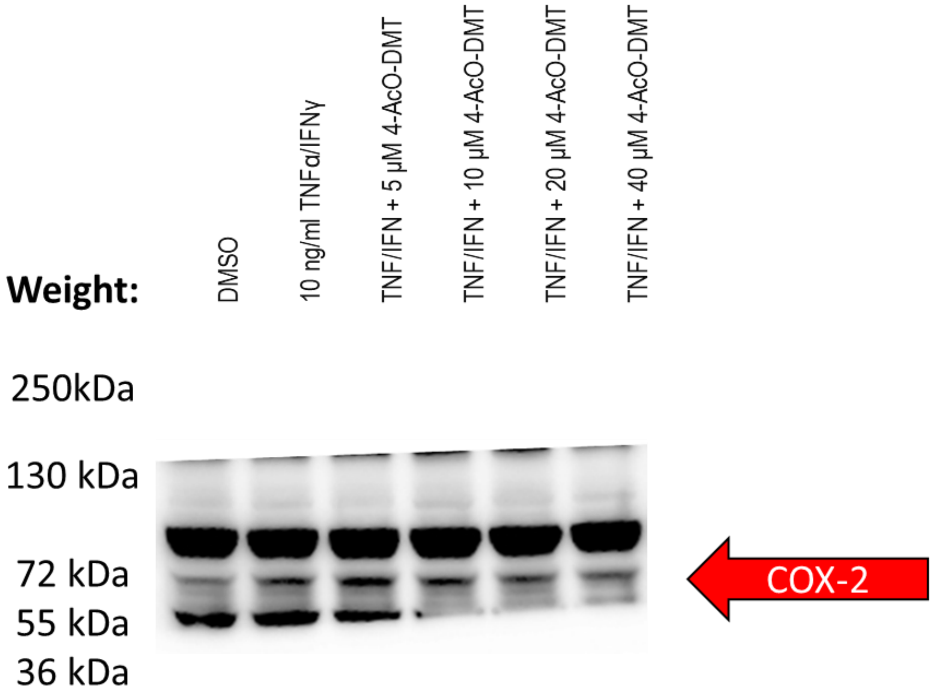

Figure S3. Original Western blots of HSEIC proteins showing COX-2 at molecular weight of 72 kDa. Red arrow indicates bands shown in Figure 6B. This blot membrane has been cut into pieces. Each membrane received different exposure times, and the images were taken separately.

GAPDH (36 kDa) in Figure 6B

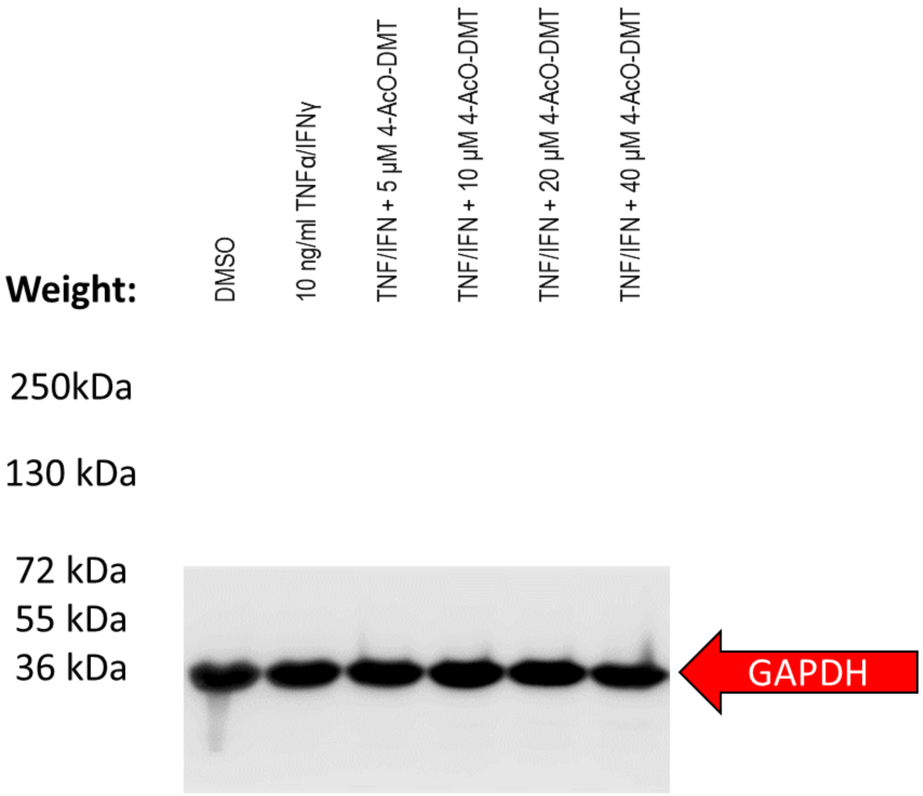

Figure S4. Original Western blots of HSEIC proteins showing GAPDH at molecular weight of 36 kDa. Red arrow indicates bands shown in Figure 6B. This blot membrane has been cut into pieces. Each membrane received different exposure times, and the images were taken separately.

COX-2 (72 kDa) in Figure 6C

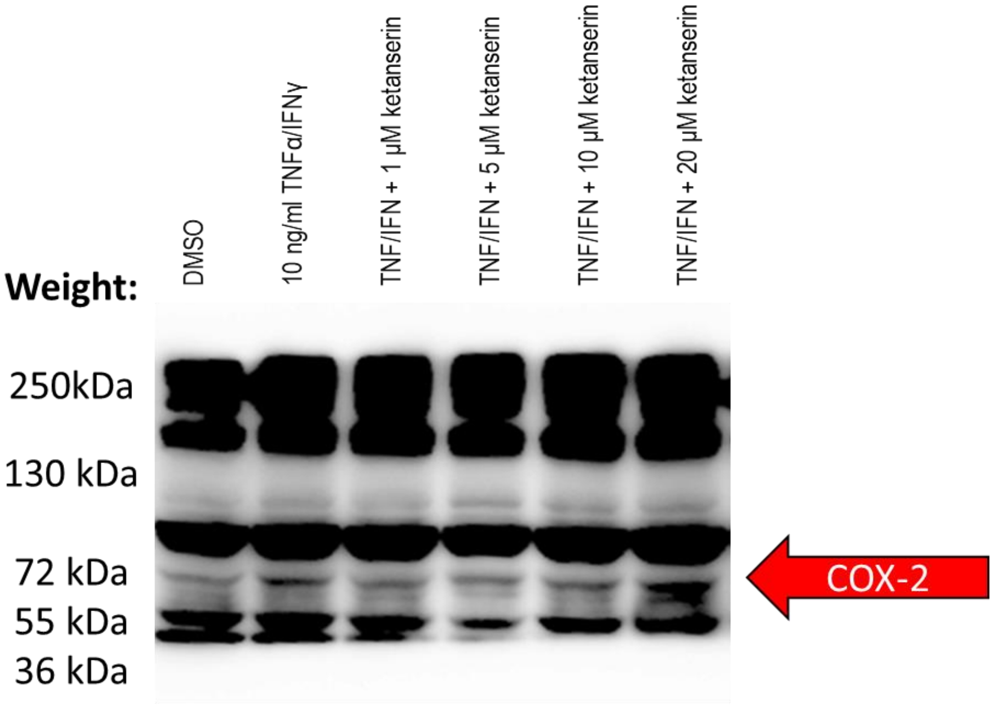

Figure S5. Original Western blots of HSEIC proteins showing COX-2 at molecular weight of 72 kDa. Red arrow indicates bands shown in Figure 6C.

GAPDH (36 kDa) in Figure 6C

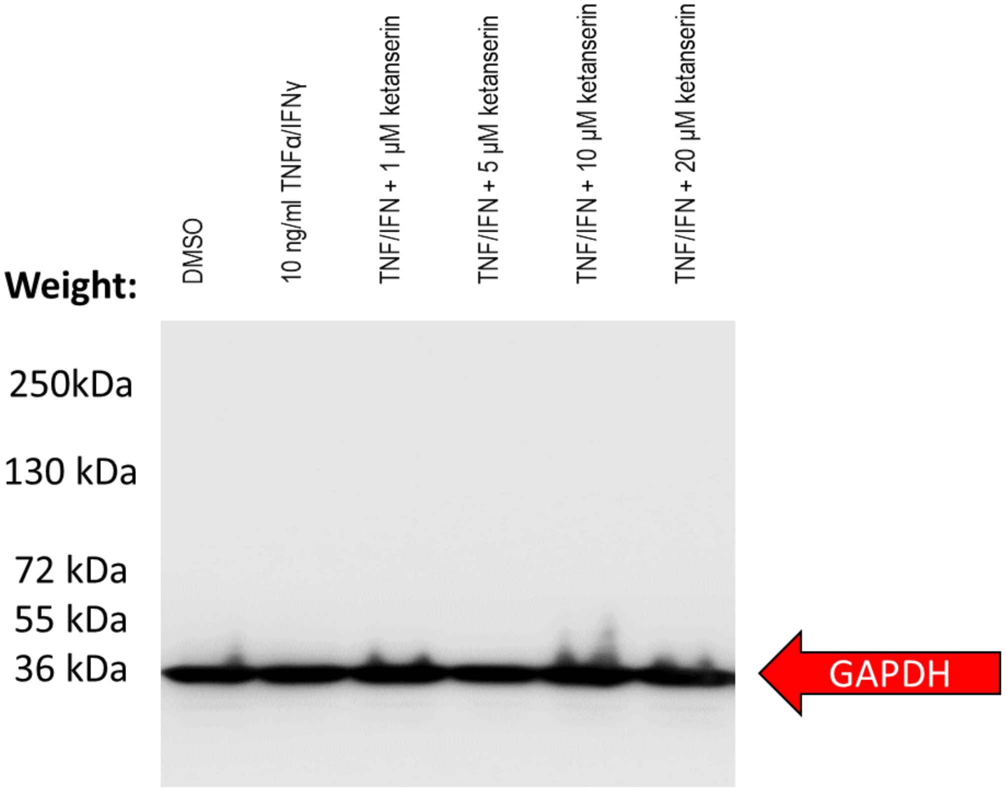

Figure S6. Original Western blots of HSEIC proteins showing GAPDH at molecular weight of 36 kDa. Red arrow indicates bands shown in Figure 6C.

COX-2 (72 kDa) in Figure 6D

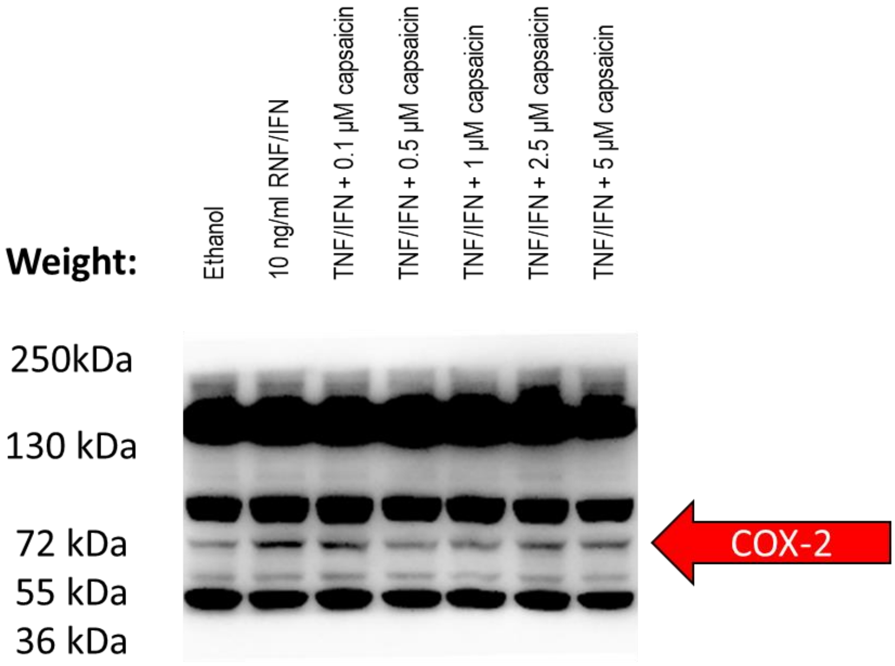

Figure S7. Original Western blots of HSEIC proteins showing COX-2 at molecular weight of 72 kDa. Red arrow indicates bands shown in Figure 6D.

GAPDH (36 kDa) in Figure 6D

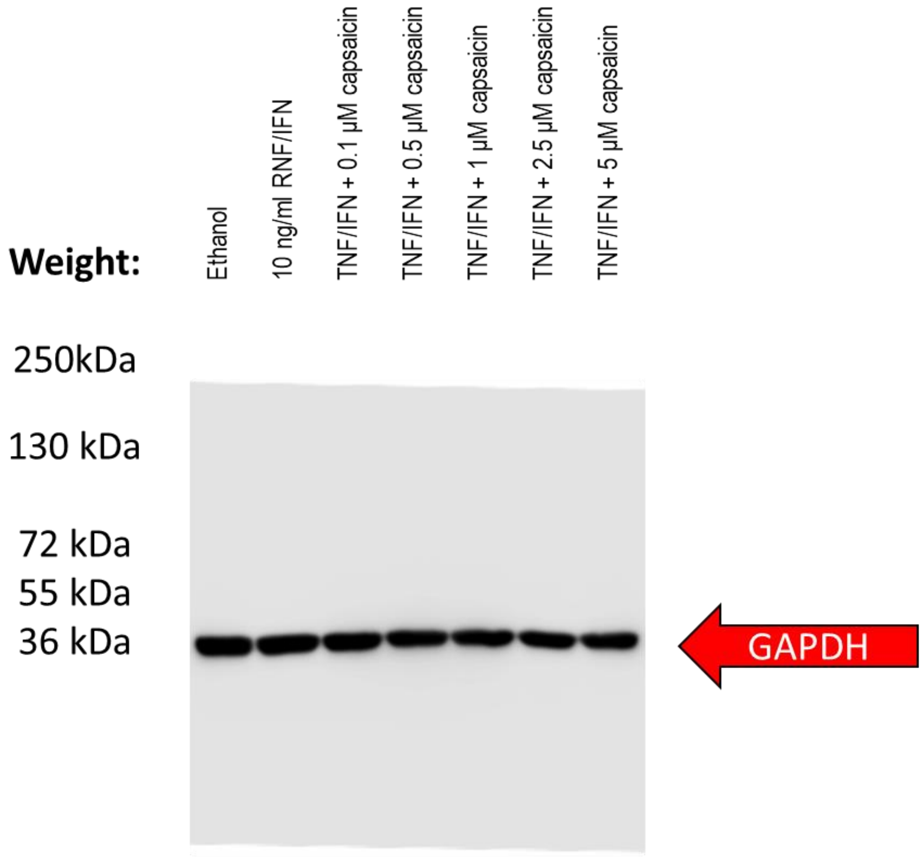

Figure S8. Original Western blots of HSEIC proteins showing GAPDH at molecular weight of 36 kDa. Red arrow indicates bands shown in Figure 6D.

COX-2 (72 kDa) in Figure 6E

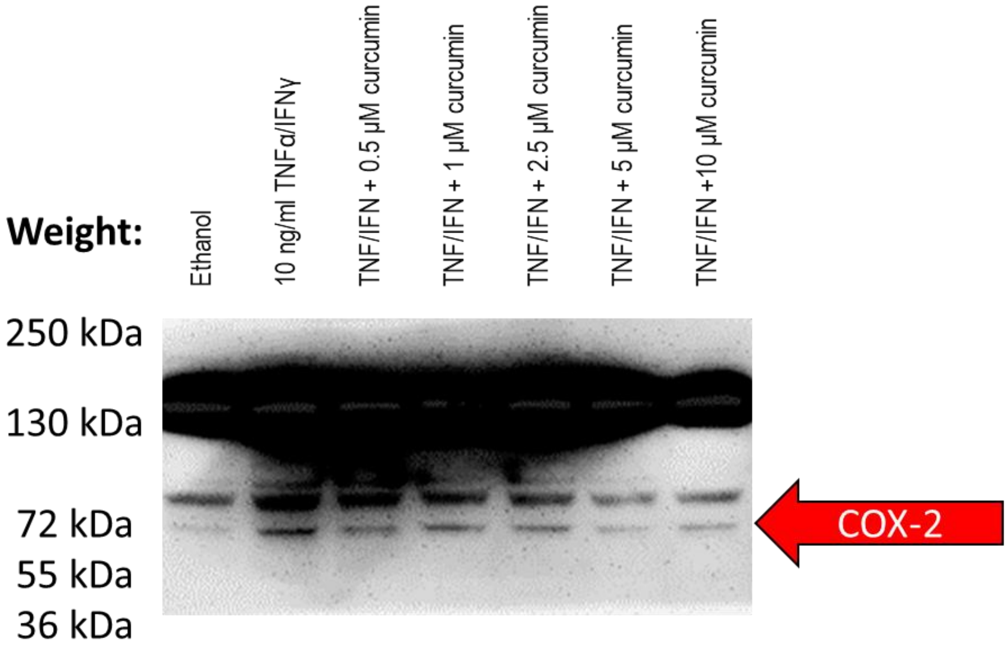

Figure S9. Original Western blots of HSEIC proteins showing COX-2 at molecular weight of 72 kDa. Red arrow indicates bands shown in Figure 6E. This blot membrane has been cut into pieces. Each membrane received different exposure times, and the images were taken separately.

GAPDH (36 kDa) in Figure 6E

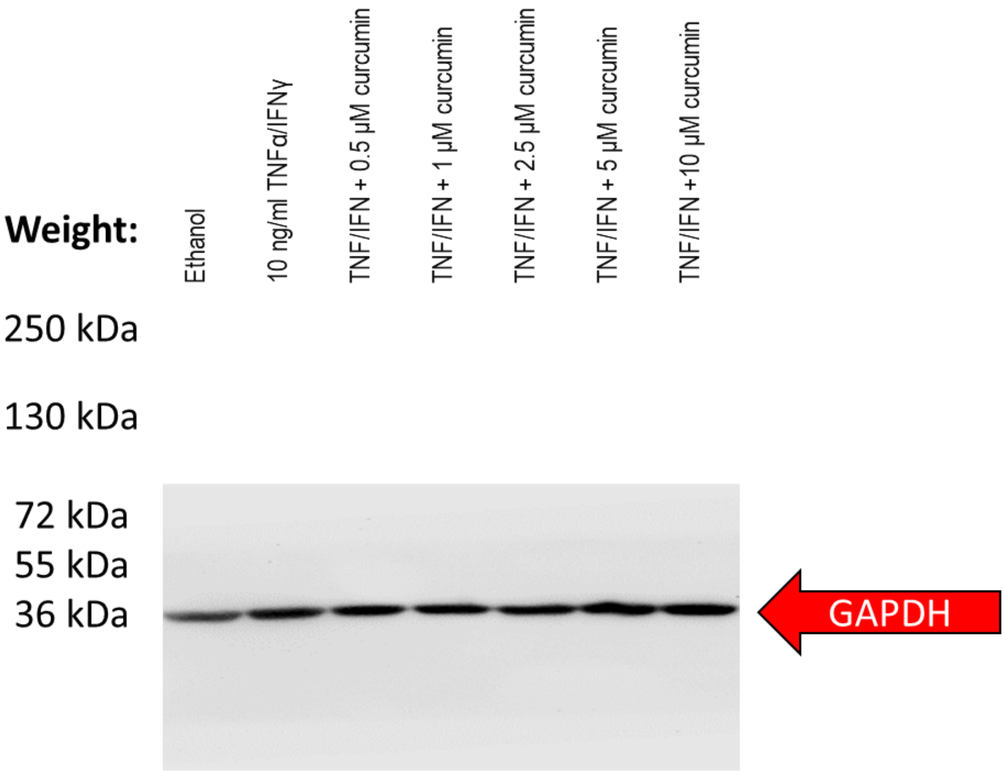

Figure S10. Original Western blots of HSEIC proteins showing GAPDH at molecular weight of 36 kDa. Red arrow indicates bands shown in Figure 6E. This blot membrane has been cut into pieces. Each membrane received different exposure times, and the images were taken separately.

COX-2 (72 kDa) in Figure 6F

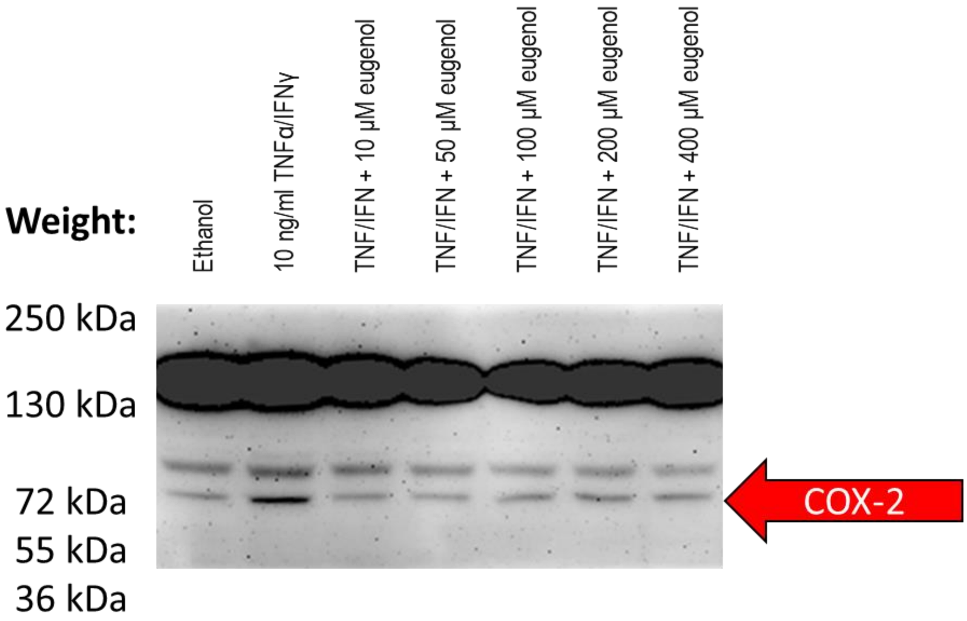

Figure S11. Original Western blots of HSEIC proteins showing COX-2 at molecular weight of 72 kDa. Red arrow indicates bands shown in Figure 6F. This blot membrane has been cut into pieces. Each membrane received different exposure times, and the images were taken separately.

GAPDH (36 kDa) in Figure 6F

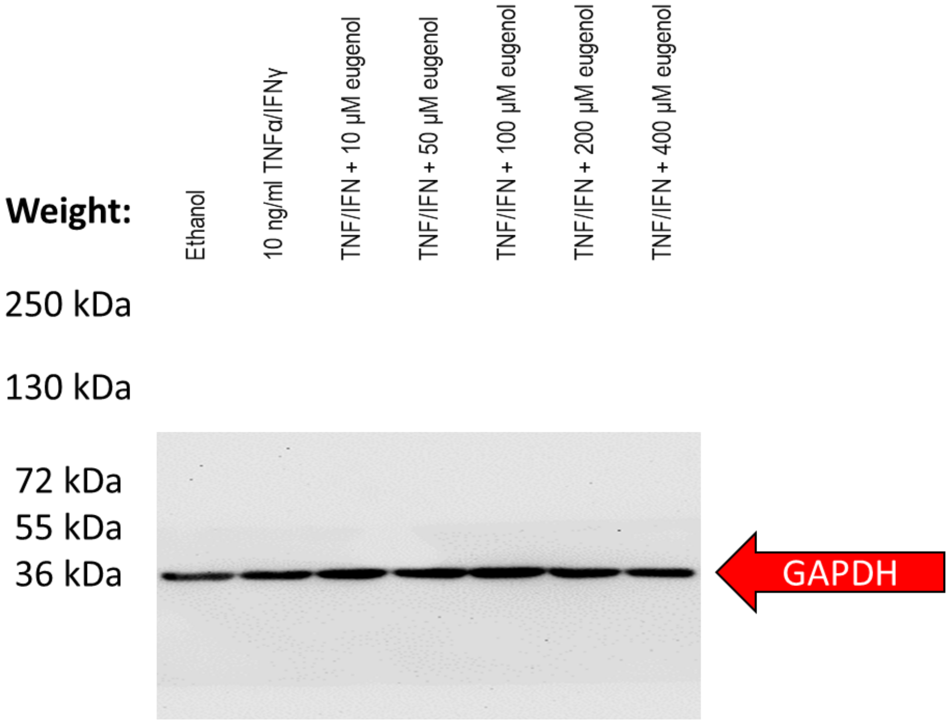

Figure S12. Original Western blots of HSEIC proteins showing GAPDH at molecular weight of 36 kDa. Red arrow indicates bands shown in Figure 6F. This blot membrane has been cut into pieces. Each membrane received different exposure times, and the images were taken separately.

COX-2 (72 kDa) in Figure 7C

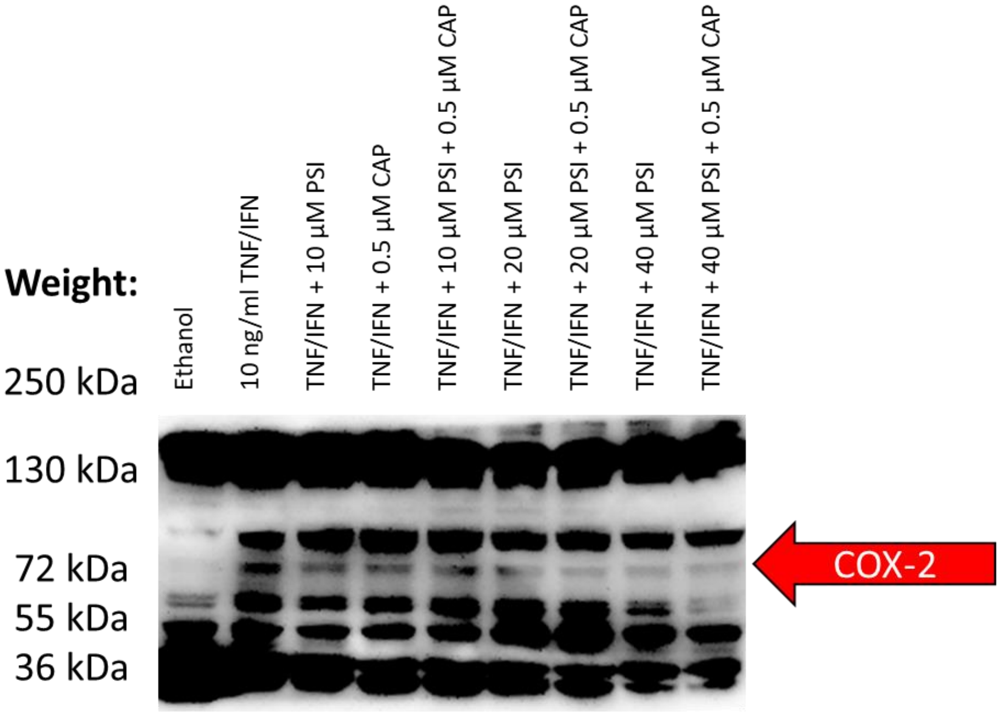

Figure S13. Original Western blots of HSEIC proteins showing COX-2 at molecular weight of 72 kDa. Red arrow indicates bands shown in Figure 7C. This blot membrane has been cut into pieces. Each membrane received different exposure times, and the images were taken separately.

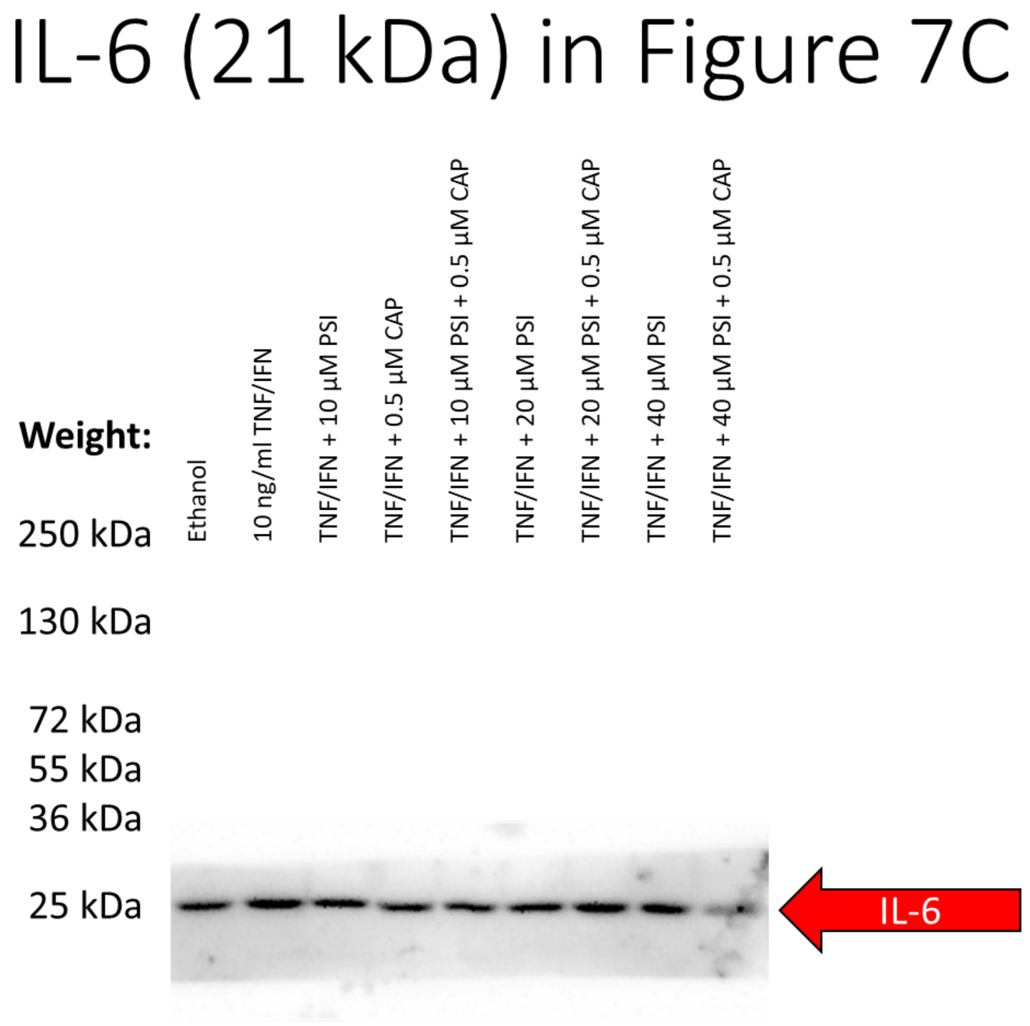

Figure S14. Original Western blots of HSEIC proteins showing IL-6 at molecular weight of 21 kDa. Red arrow indicates bands shown in Figure 7C. This blot membrane has been cut into pieces. Each membrane received different exposure times, and the images were taken separately.

GAPDH (36 kDa) in Figure 7C

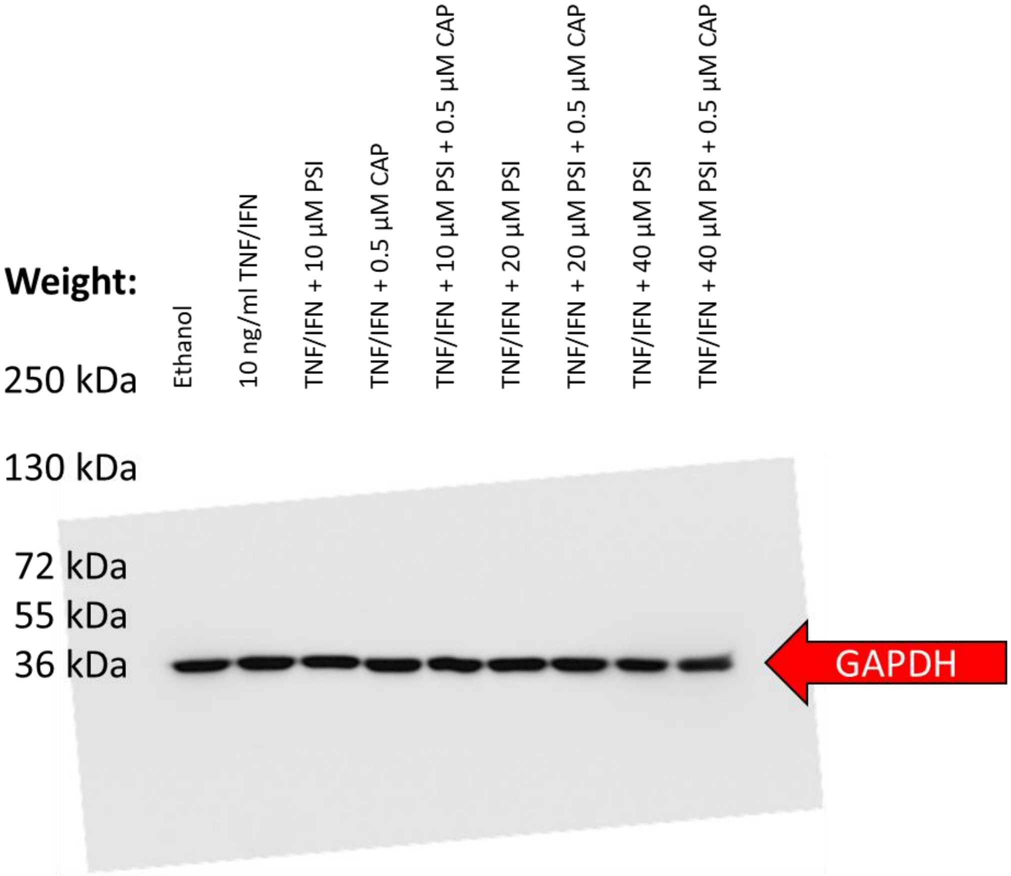

Figure S15. Original Western blots of HSEIC proteins showing GAPDH at molecular weight of 36 kDa. Red arrow indicates bands shown in Figure 7C. This blot membrane has been cut into pieces. Each membrane received different exposure times, and the images were taken separately.

COX-2 (72 kDa) in Figure 8C

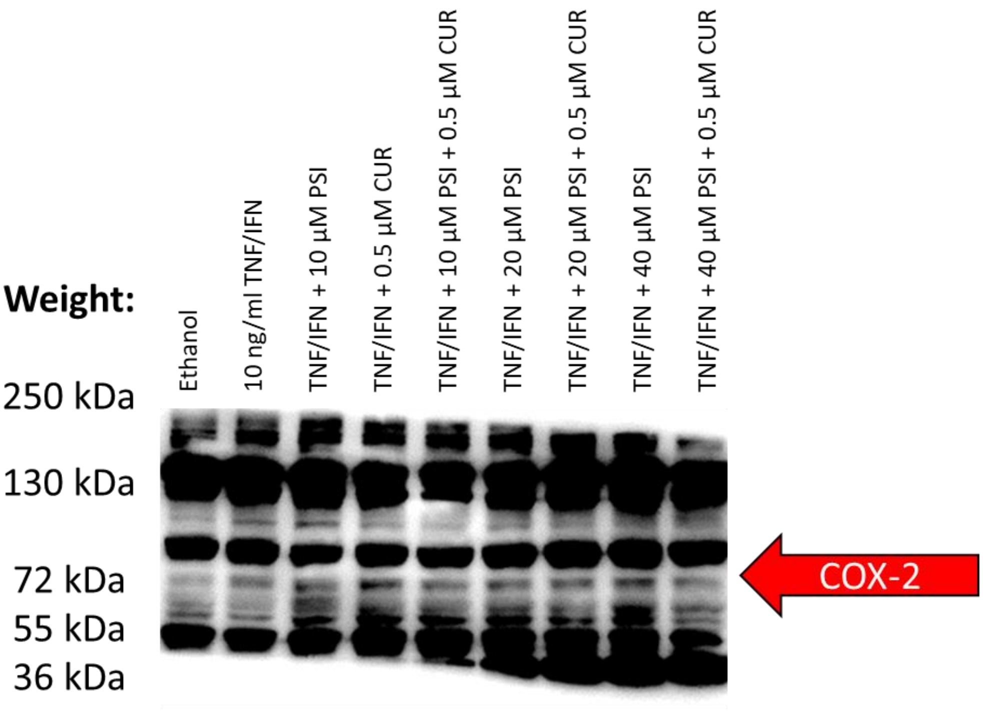

Figure S16. Original Western blots of HSEIC proteins showing COX-2 at molecular weight of 72 kDa. Red arrow indicates bands shown in Figure 8C. This blot membrane has been cut into pieces. Each membrane received different exposure times, and the images were taken separately.

IL-6 (25 kDa) in Figure 8C

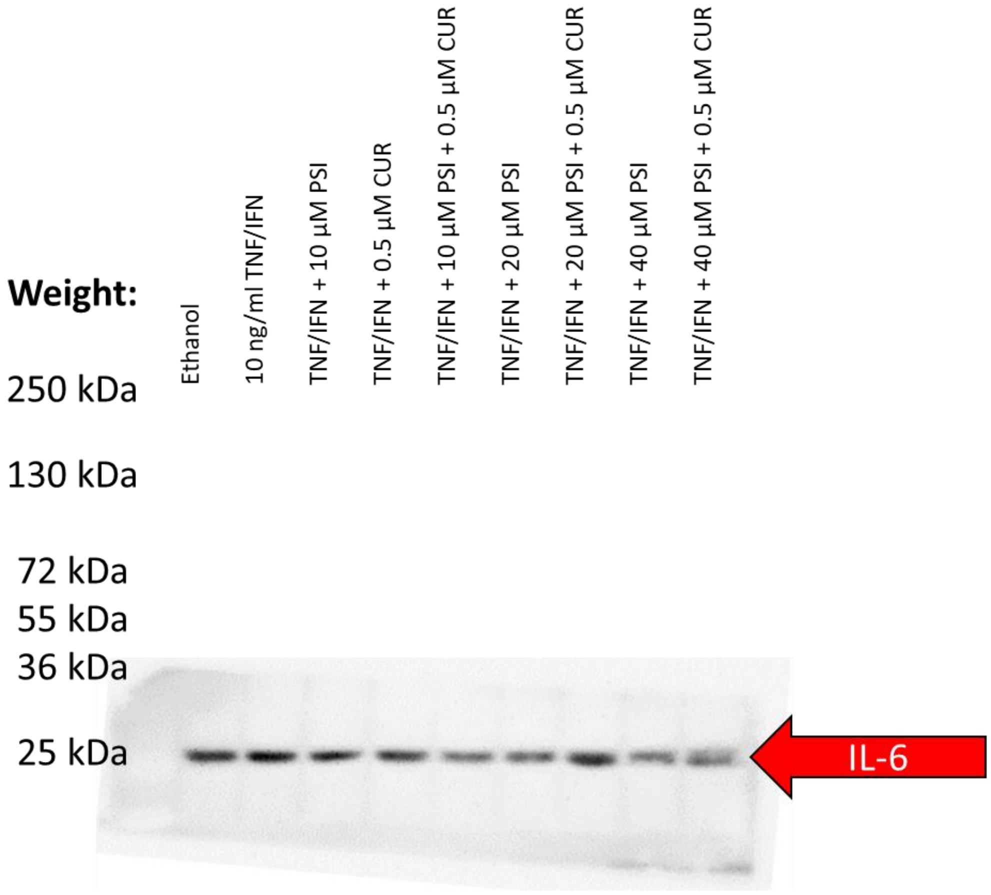

Figure S17. Original Western blots of HSEIC proteins showing IL-6 at molecular weight of 21 kDa. Red arrow indicates bands shown in Figure 8C. This blot membrane has been cut into pieces. Each membrane received different exposure times, and the images were taken separately.

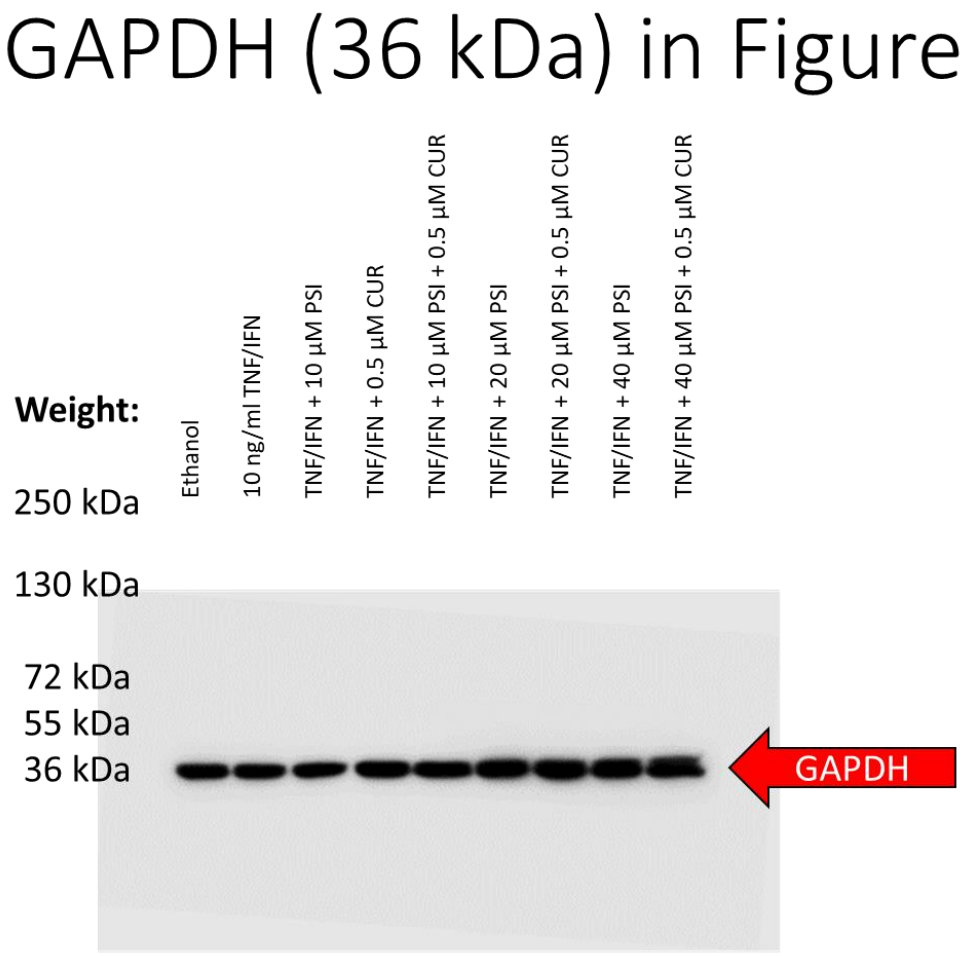

Figure S18. Original Western blots of HSEIC proteins showing GAPDH at molecular weight of 36 kDa. Red arrow indicates bands shown in Figure 8C. This blot membrane has been cut into pieces. Each membrane received different exposure times, and the images were taken separately.

COX-2 (72 kDa) in Figure 9C

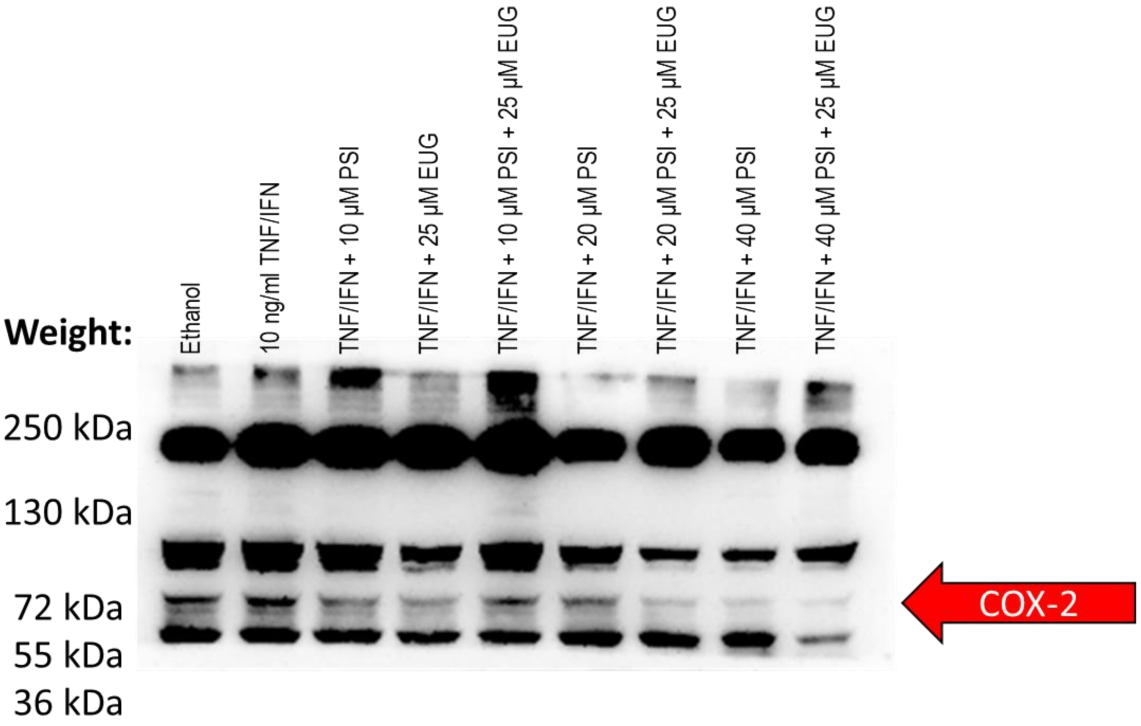

Figure S19. Original Western blots of HSEIC proteins showing COX-2 at molecular weight of 72 kDa. Red arrow indicates bands shown in Figure 9C. This blot membrane has been cut into pieces. Each membrane received different exposure times, and the images were taken separately.

IL-6 (25 kDa) in Figure 9C

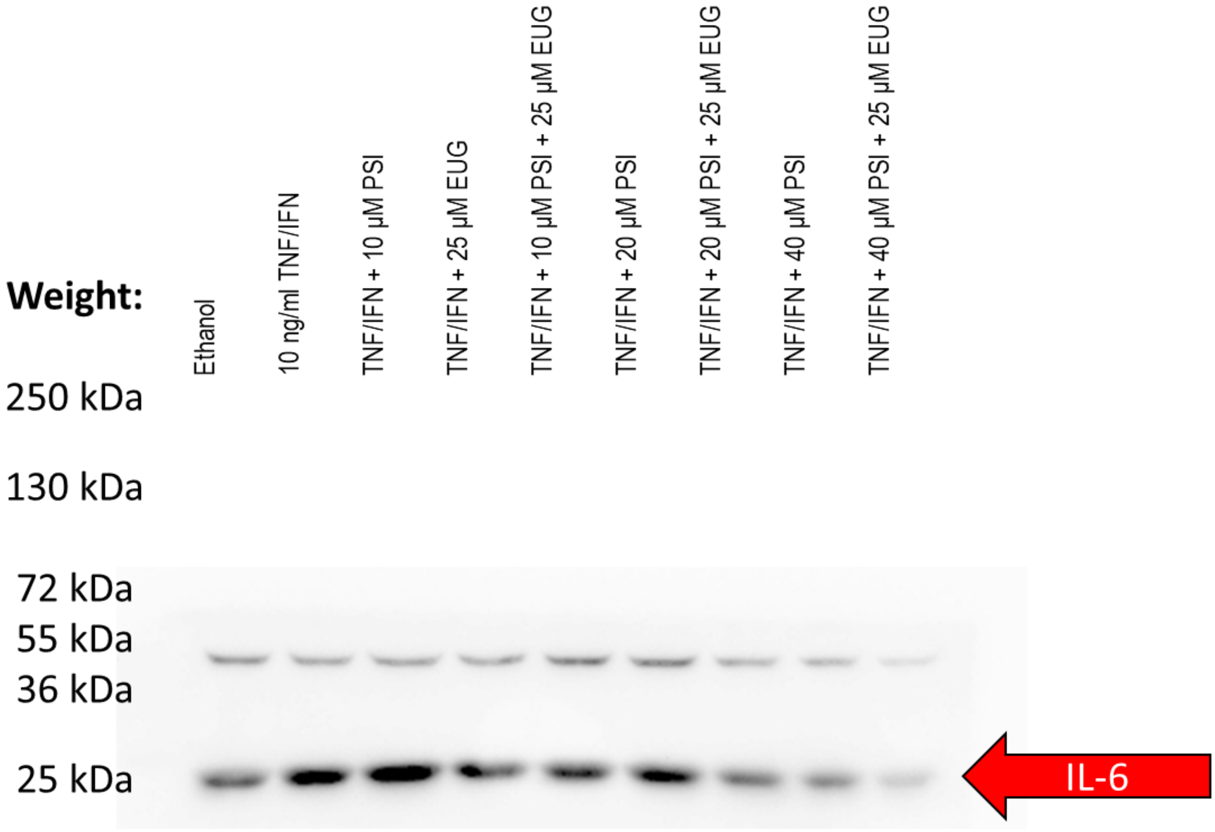

Figure S20. Original Western blots of HSEIC proteins showing IL-6 at molecular weight of 21 kDa. Red arrow indicates bands shown in Figure 9C. This blot membrane has been cut into pieces. Each membrane received different exposure times, and the images were taken separately.

GAPDH (36 kDa) in Figure 9C

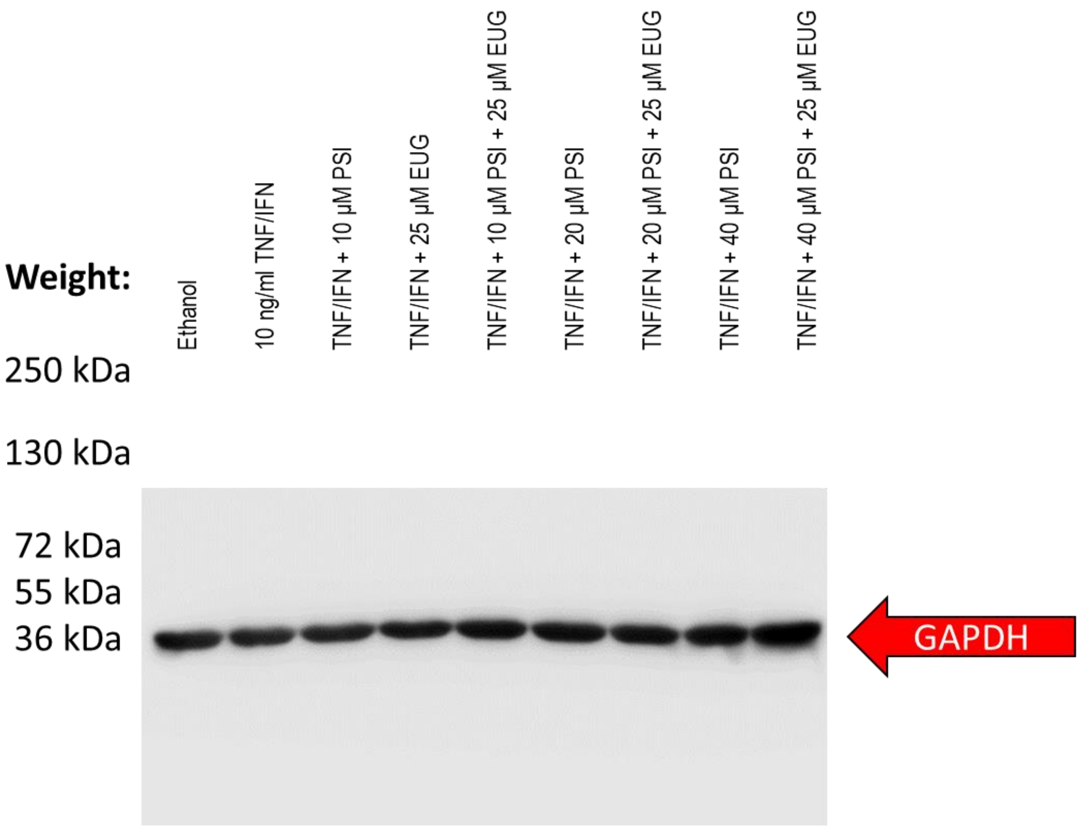

Figure S21. Original Western blots of HSEIC proteins showing GAPDH at molecular weight of 36 kDa. Red arrow indicates bands shown in Figure 9C. This blot membrane has been cut into pieces. Each membrane received different exposure times, and the images were taken separately.

COX-2 (72 kDa) in Figure 10C

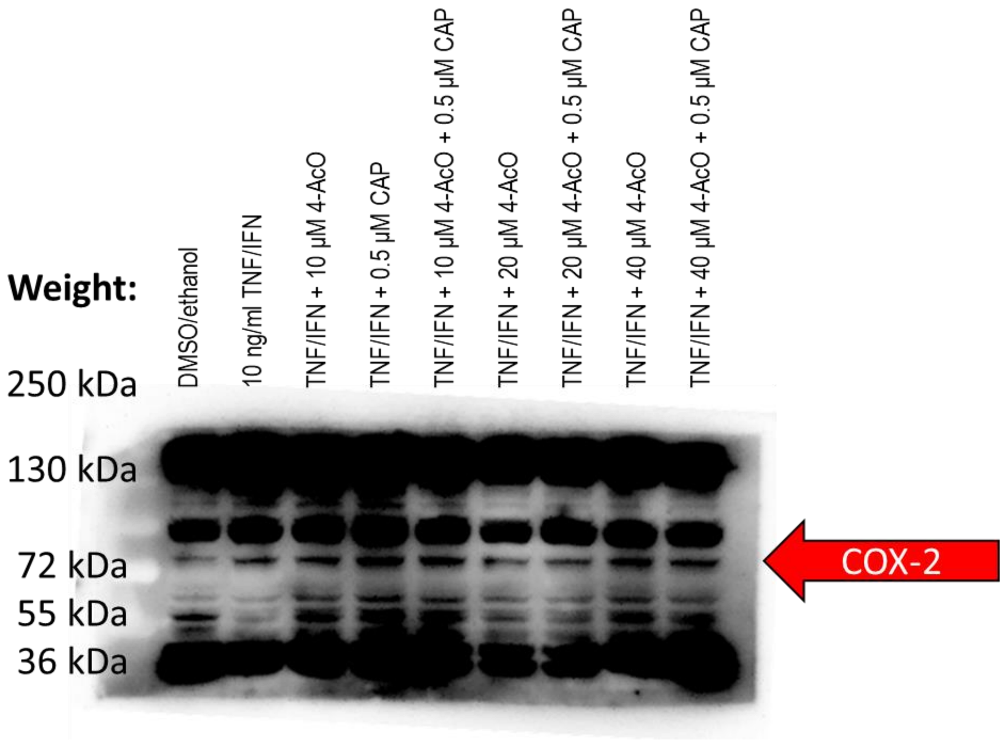

Figure S22. Original Western blots of HSEIC proteins showing COX-2 at molecular weight of 72 kDa. Red arrow indicates bands shown in Figure 10C. This blot membrane has been cut into pieces. Each membrane received different exposure times, and the images were taken separately.

IL-6 (25 kDa) in Figure 10C

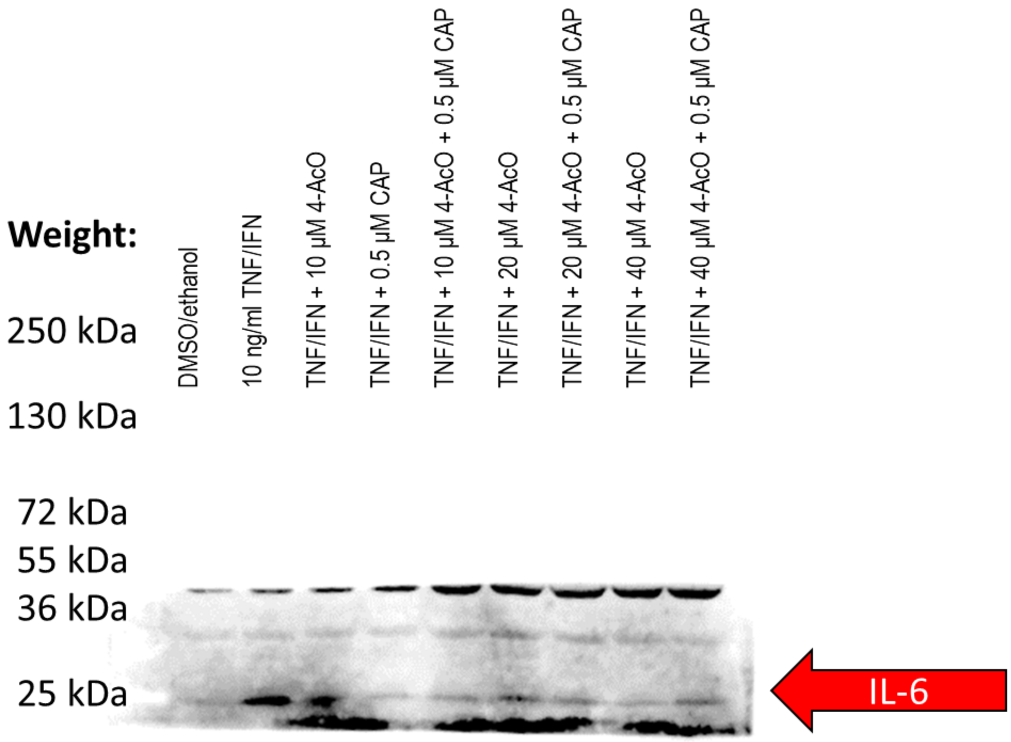

Figure S23. Original Western blots of HSEIC proteins showing IL-6 at molecular weight of 21 kDa. Red arrow indicates bands shown in Figure 10C. This blot membrane has been cut into pieces. Each membrane received different exposure times, and the images were taken separately.

GAPDH (36 kDa) in Figure 10C

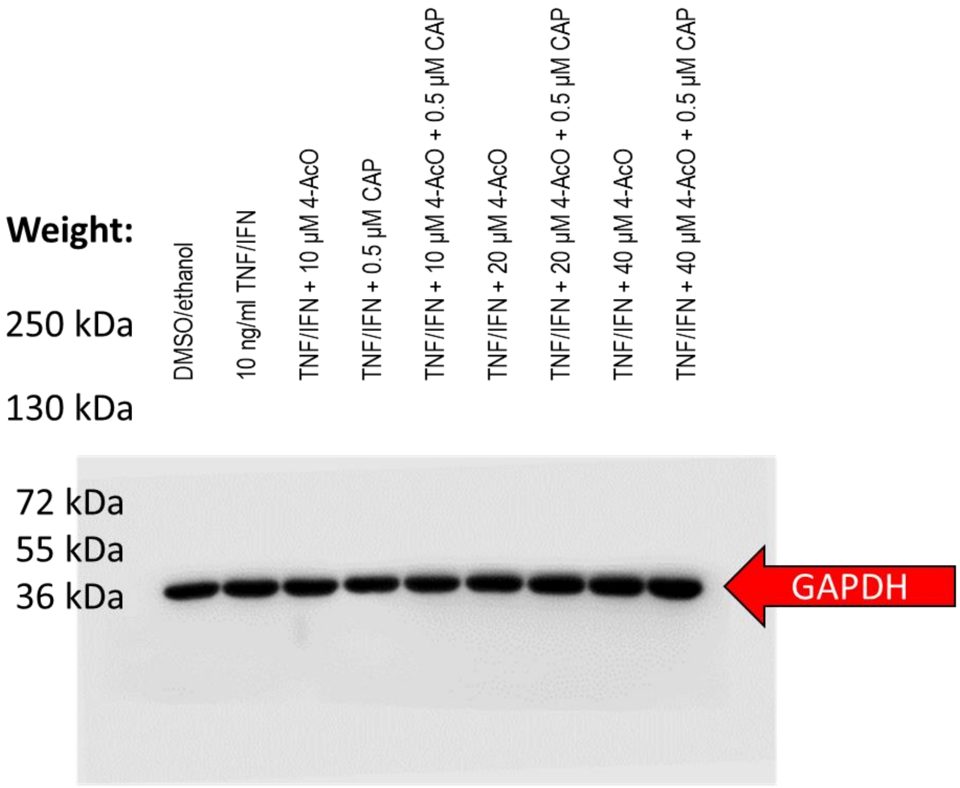

Figure S24. Original Western blots of HSEIC proteins showing GAPDH at molecular weight of 36 kDa. Red arrow indicates bands shown in Figure 10C. This blot membrane has been cut into pieces. Each membrane received different exposure times, and the images were taken separately.

COX-2 (72 kDa) in Figure 11C

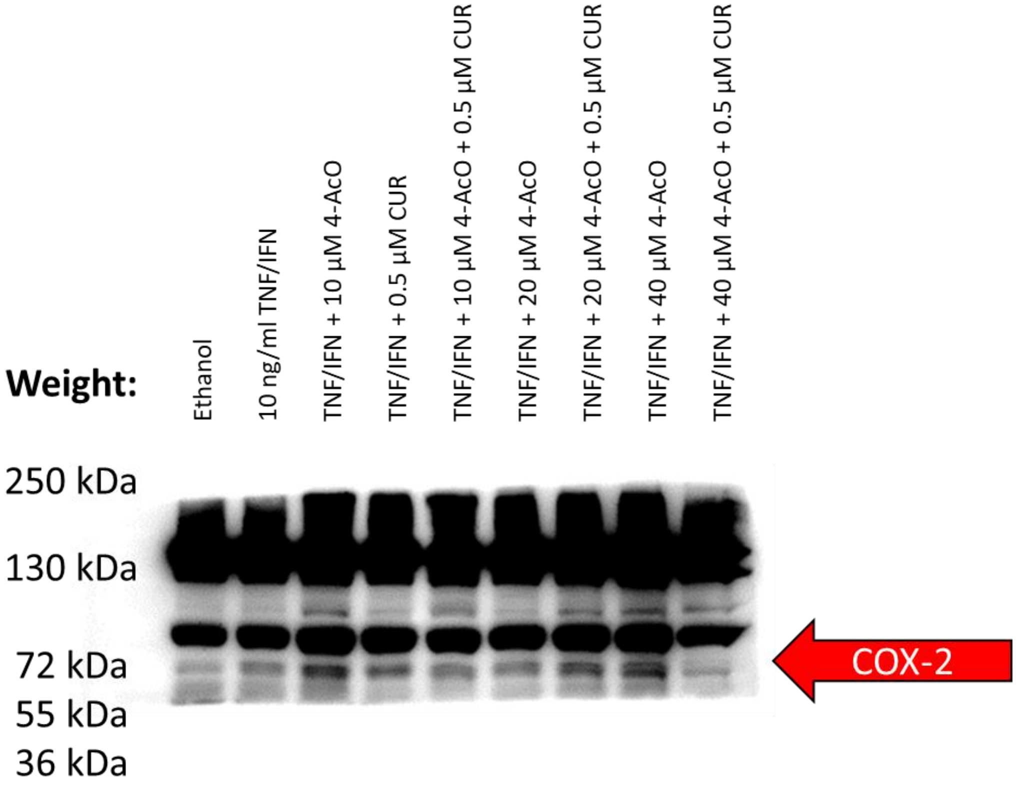

Figure S25. Original Western blots of HSEIC proteins showing COX-2 at molecular weight of 72 kDa. Red arrow indicates bands shown in Figure 11C. This blot membrane has been cut into pieces. Each membrane received different exposure times, and the images were taken separately.

IL-6 (25 kDa) in Figure 11C

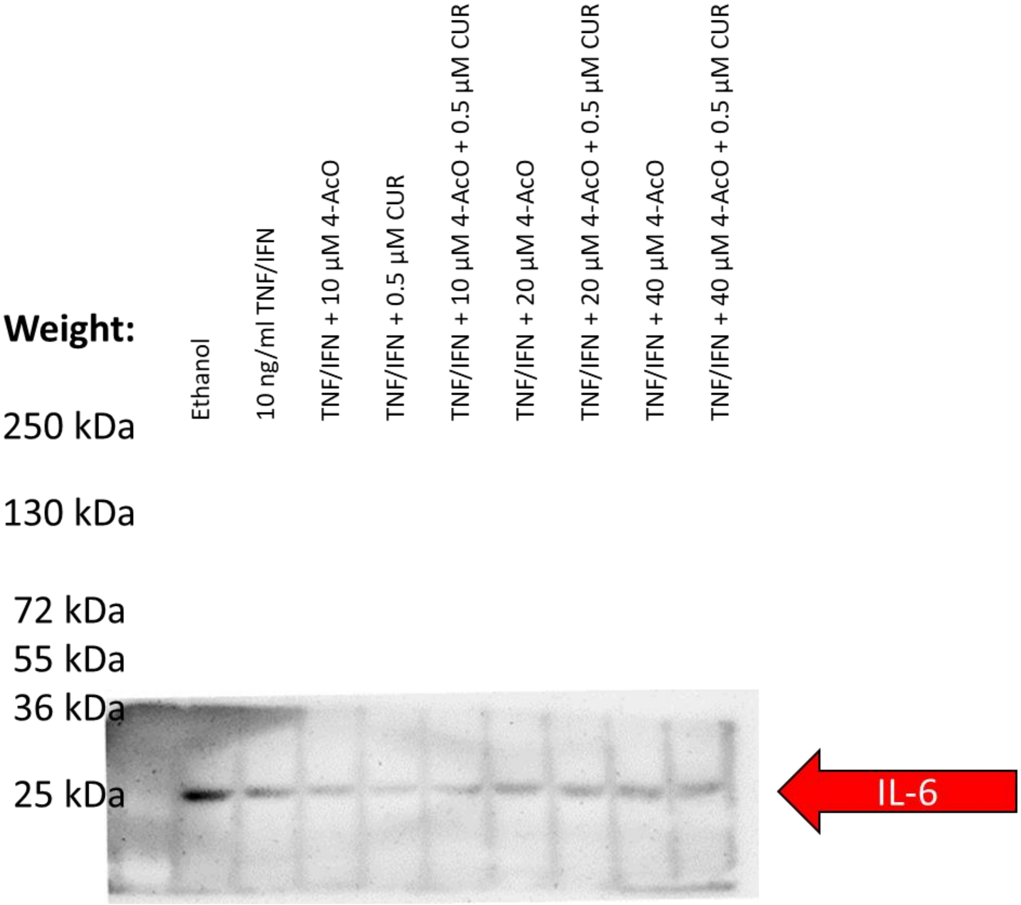

Figure S26. Original Western blots of HSEIC proteins showing IL-6 at molecular weight of 21 kDa. Red arrow indicates bands shown in Figure 11C. This blot membrane has been cut into pieces. Each membrane received different exposure times, and the images were taken separately.

GAPDH (36 kDa) in Figure 11C

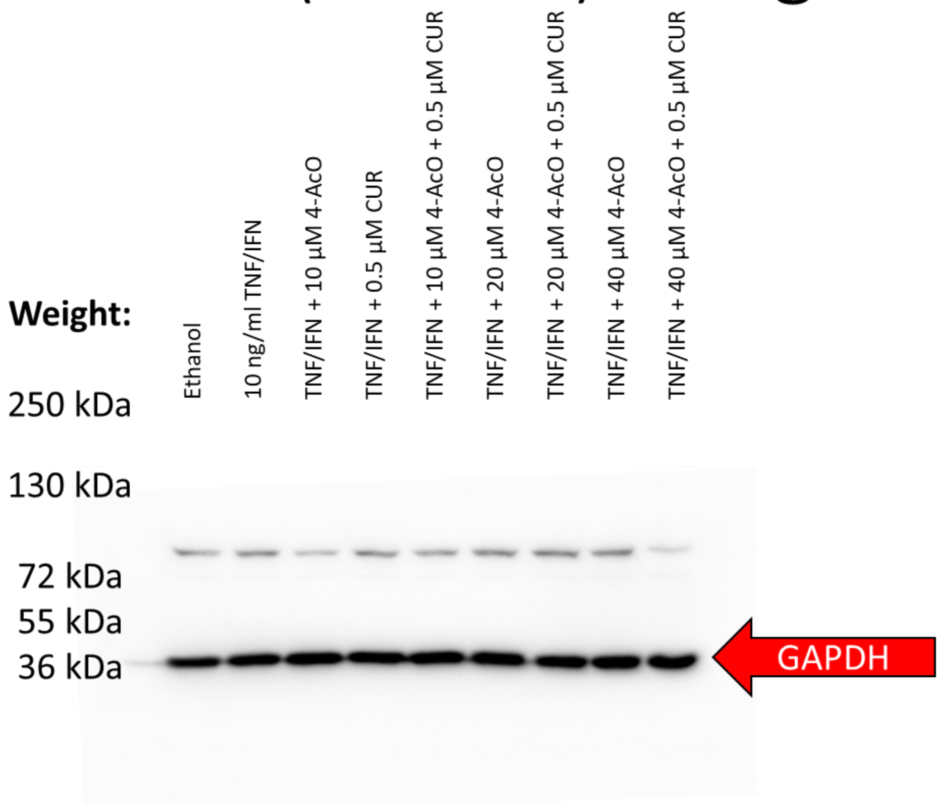

Figure S27. Original Western blots of HSEIC proteins showing GAPDH at molecular weight of 36 kDa. Red arrow indicates bands shown in Figure 11C. This blot membrane has been cut into pieces. Each membrane received different exposure times, and the images were taken separately.

COX-2 (72 kDa) in Figure 12C

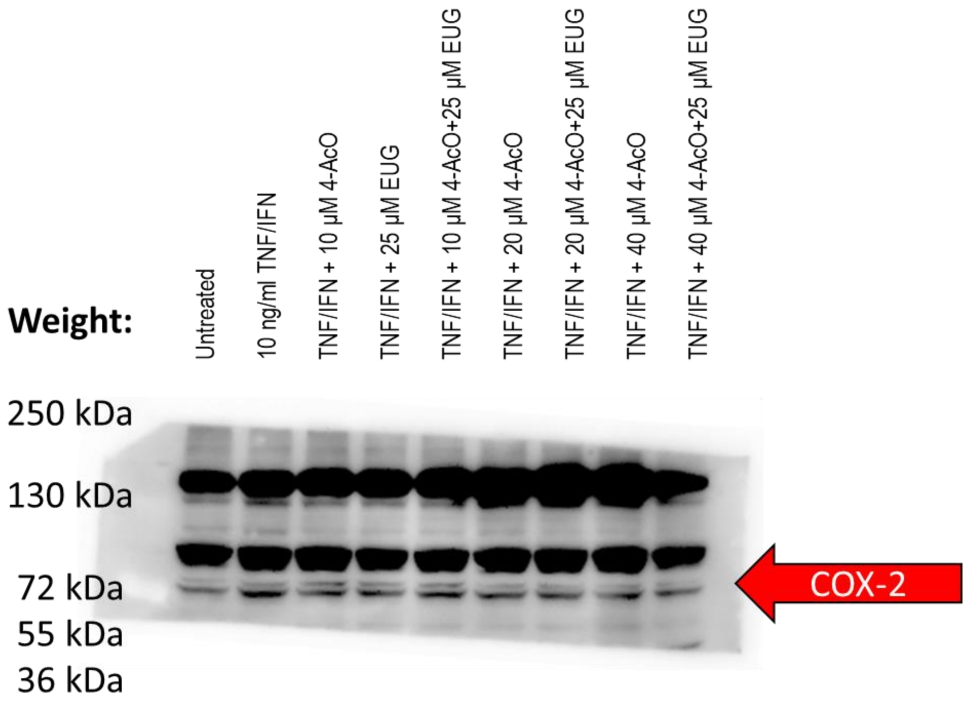

Figure S28. Original Western blots of HSEIC proteins showing COX-2 at molecular weight of 72 kDa. Red arrow indicates bands shown in Figure 12C. This blot membrane has been cut into pieces. Each membrane received different exposure times, and the images were taken separately.

IL-6 (25 kDa) in Figure 12C

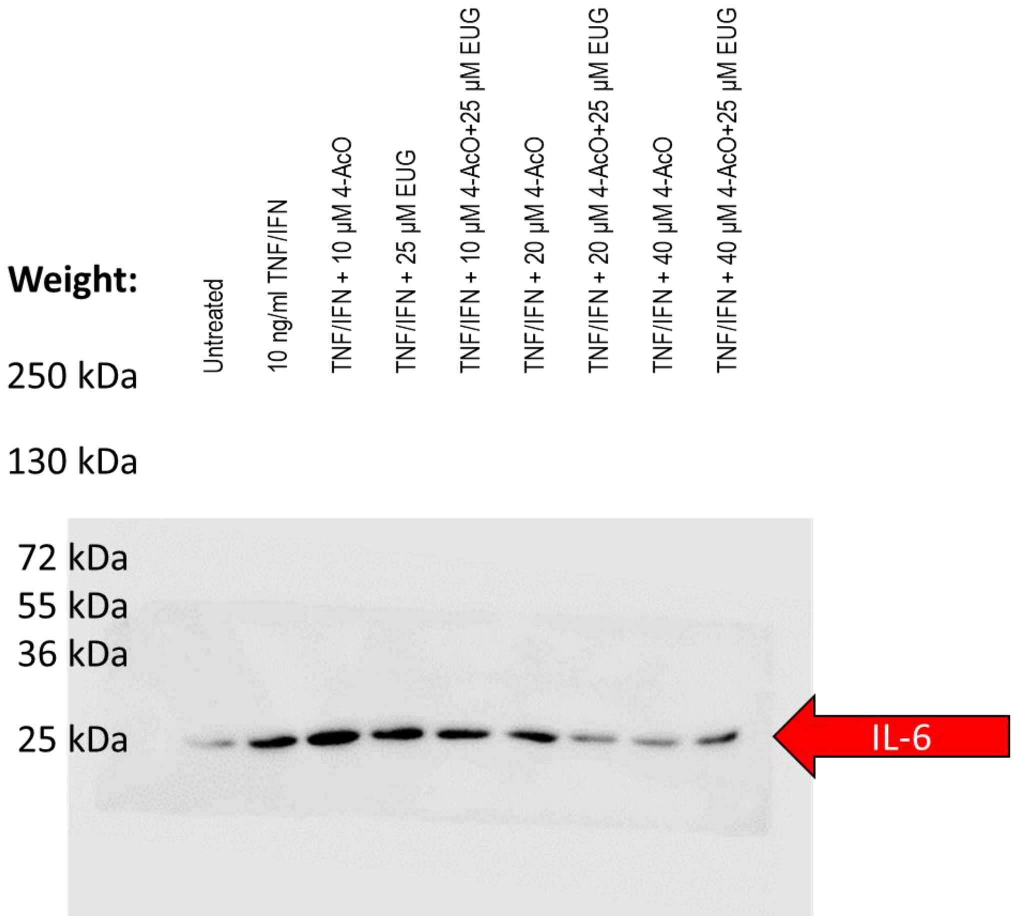

Figure S29. Original Western blots of HSEIC proteins showing IL-6 at molecular weight of 21 kDa. Red arrow indicates bands shown in Figure 12C. This blot membrane has been cut into pieces. Each membrane received different exposure times, and the images were taken separately.

GAPDH (36 kDa) in Figure 12C

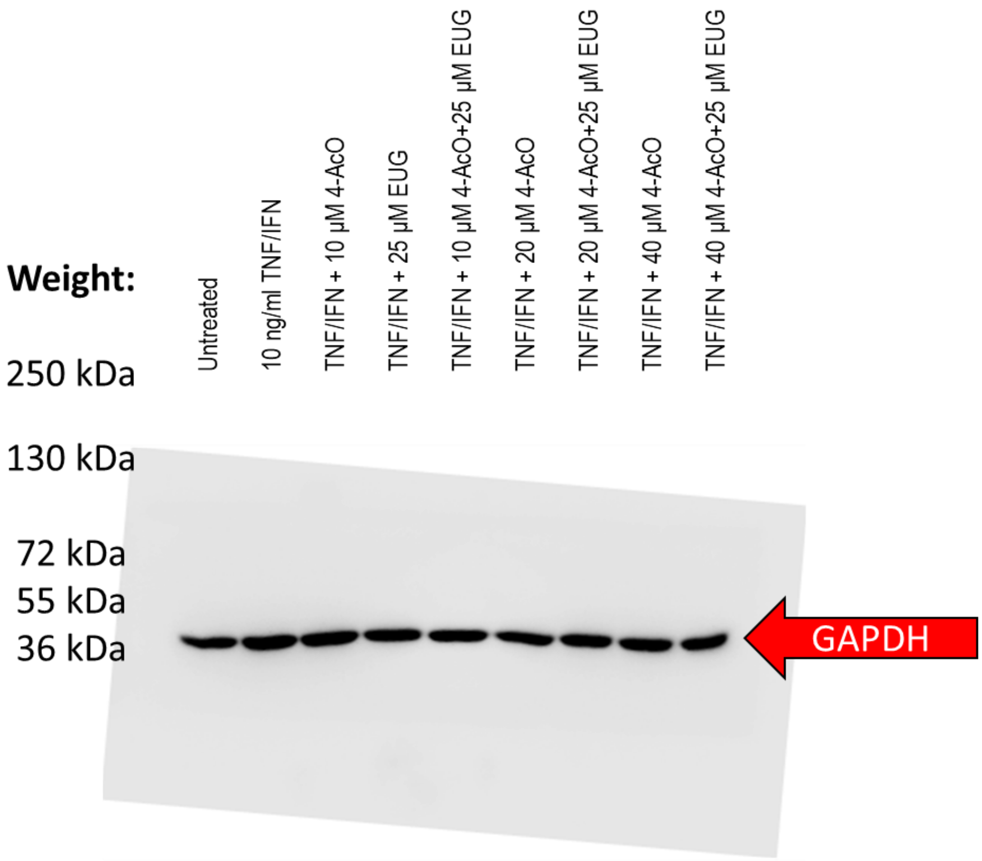

Figure S30. Original Western blots of HSEIC proteins showing GAPDH at molecular weight of 36 kDa. Red arrow indicates bands shown in Figure 12C. This blot membrane has been cut into pieces. Each membrane received different exposure times, and the images were taken separately.

COX-2 (72 kDa) in Figure 13C

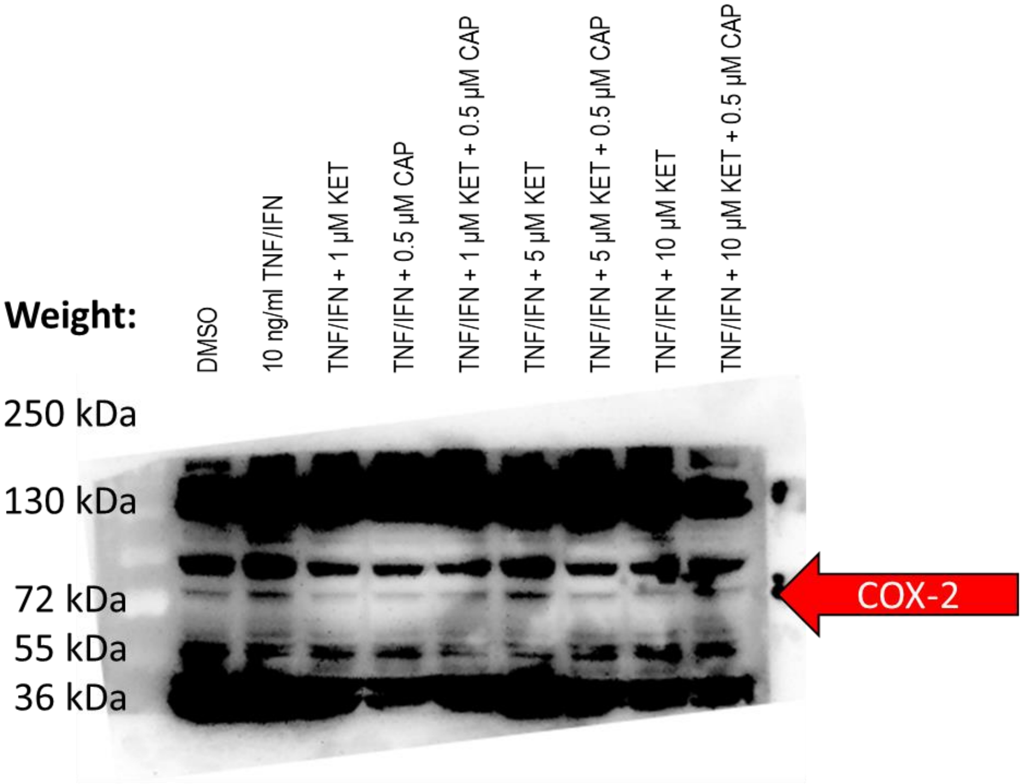

Figure S31. Original Western blots of HSEIC proteins showing COX-2 at molecular weight of 72 kDa. Red arrow indicates bands shown in Figure 13C. This blot membrane has been cut into pieces. Each membrane received different exposure times, and the images were taken separately.

IL-6 (25 kDa) in Figure 13C

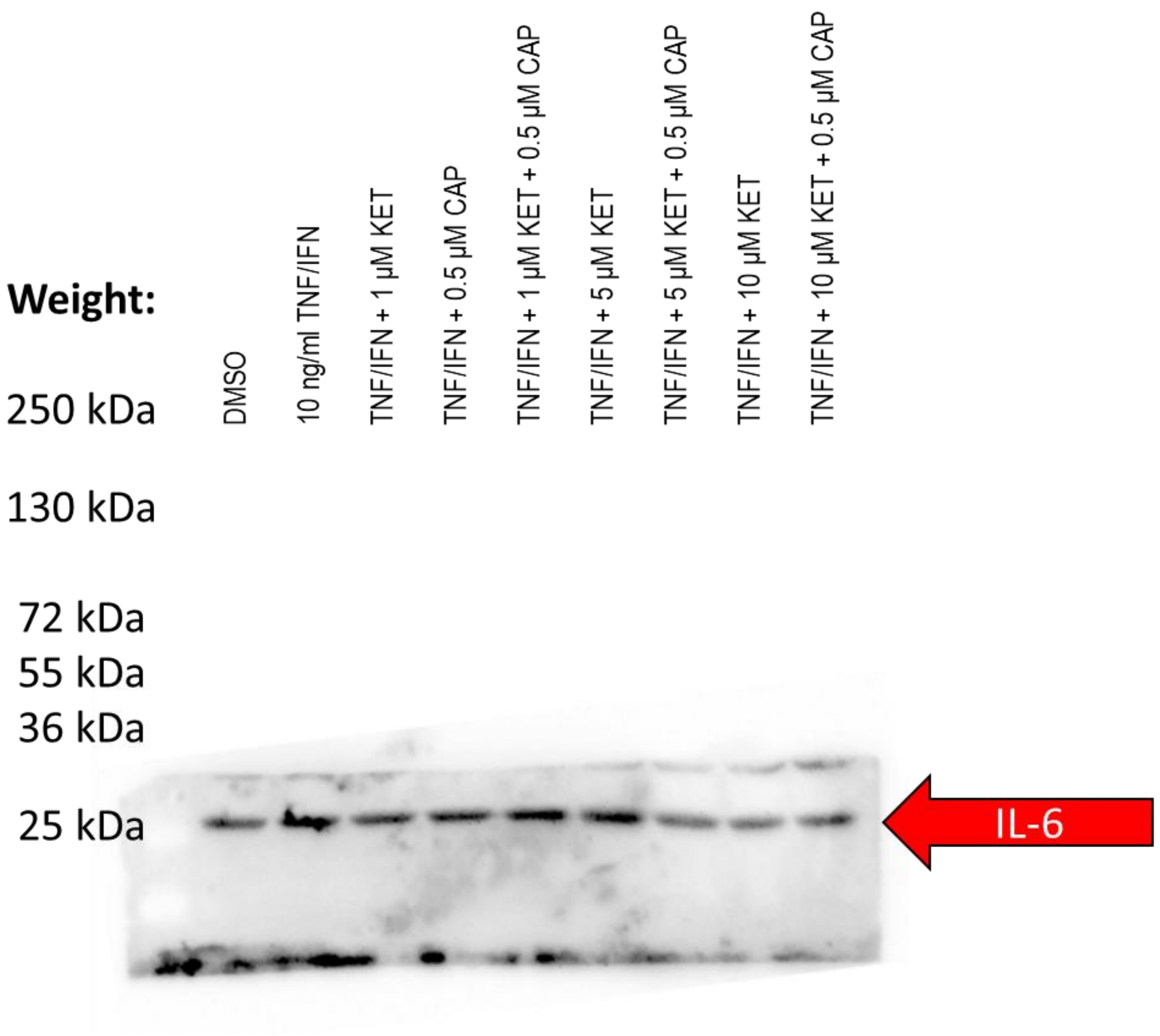

Figure S32. Original Western blots of HSEIC proteins showing IL-6 at molecular weight of 21 kDa. Red arrow indicates bands shown in Figure 13C. This blot membrane has been cut into pieces. Each membrane received different exposure times, and the images were taken separately.

GAPDH (36 kDa) in Figure 13C

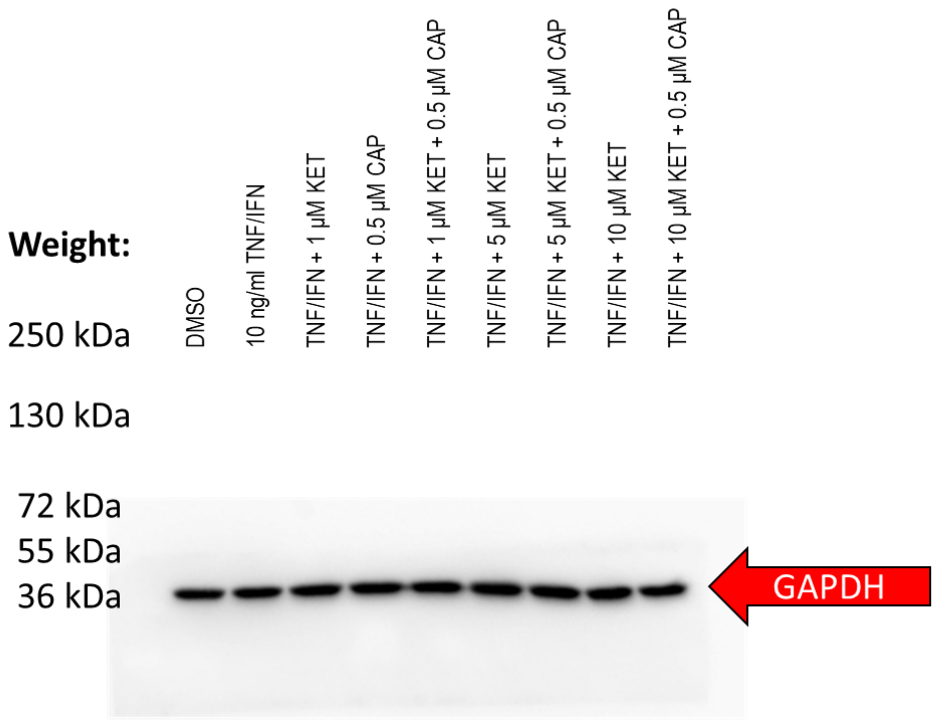

Figure S33. Original Western blots of HSEIC proteins showing GAPDH at molecular weight of 36 kDa. Red arrow indicates bands shown in Figure 13C. This blot membrane has been cut into pieces. Each membrane received different exposure times, and the images were taken separately.

COX-2 (72 kDa) in Figure 14C

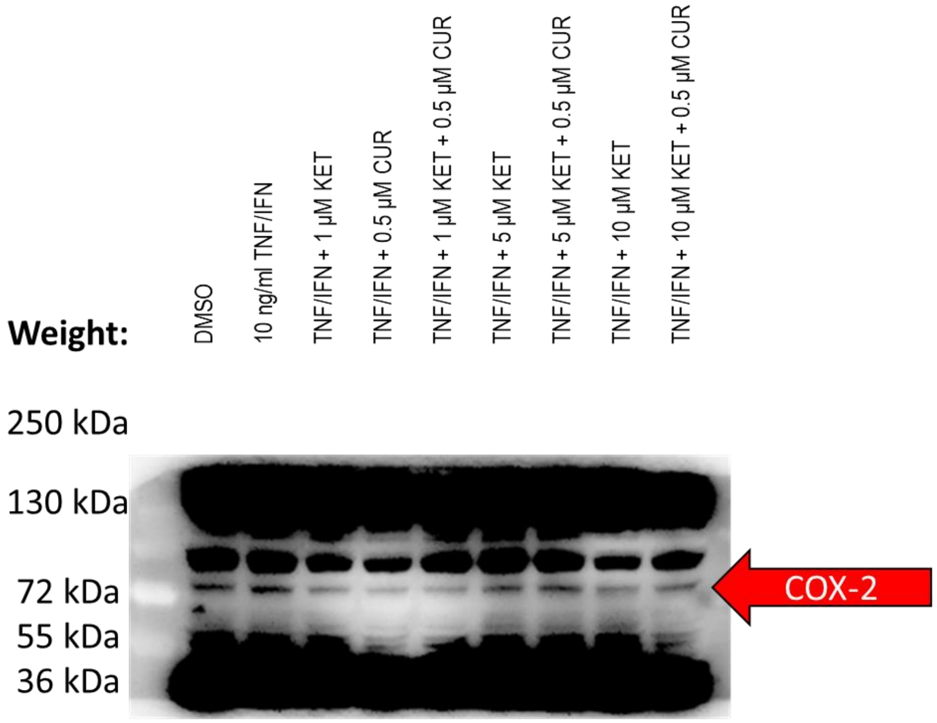

Figure S34. Original Western blots of HSEIC proteins showing COX-2 at molecular weight of 72 kDa. Red arrow indicates bands shown in Figure 14C. This blot membrane has been cut into pieces. Each membrane received different exposure times, and the images were taken separately.

IL-6 (25 kDa) in Figure 14C

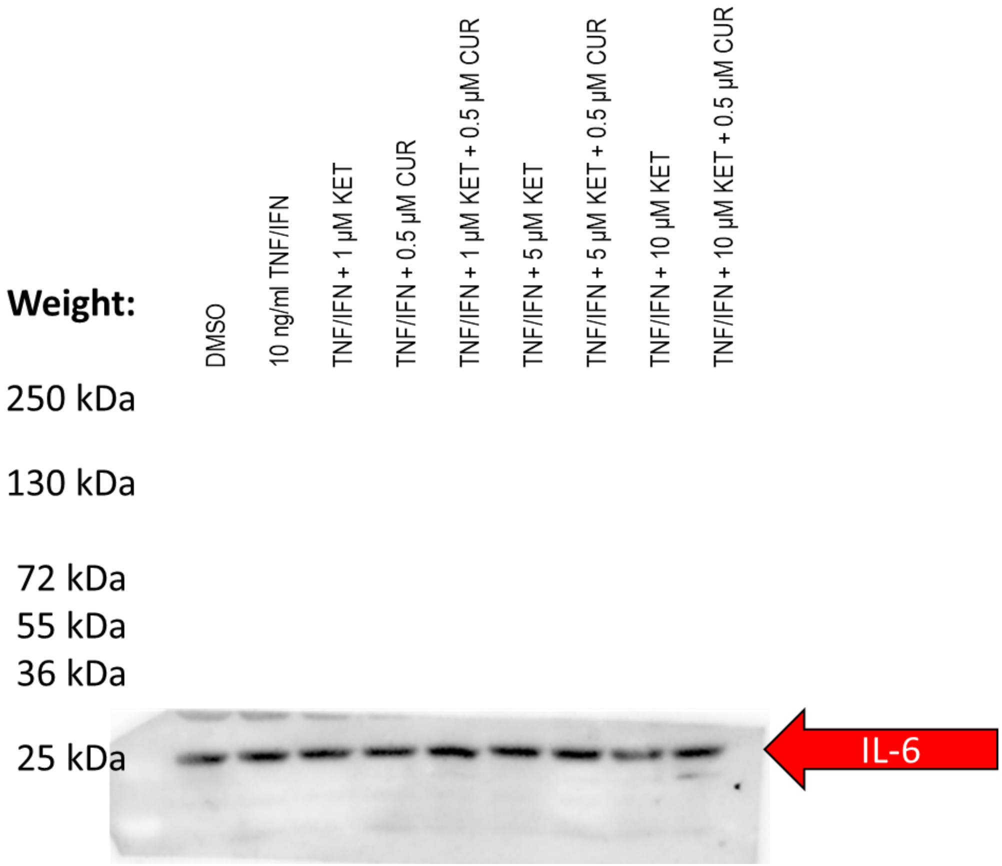

Figure S35. Original Western blots of HSEIC proteins showing IL-6 at molecular weight of 21 kDa. Red arrow indicates bands shown in Figure 14C. This blot membrane has been cut into pieces. Each membrane received different exposure times, and the images were taken separately.

GAPDH (36 kDa) in Figure 14C

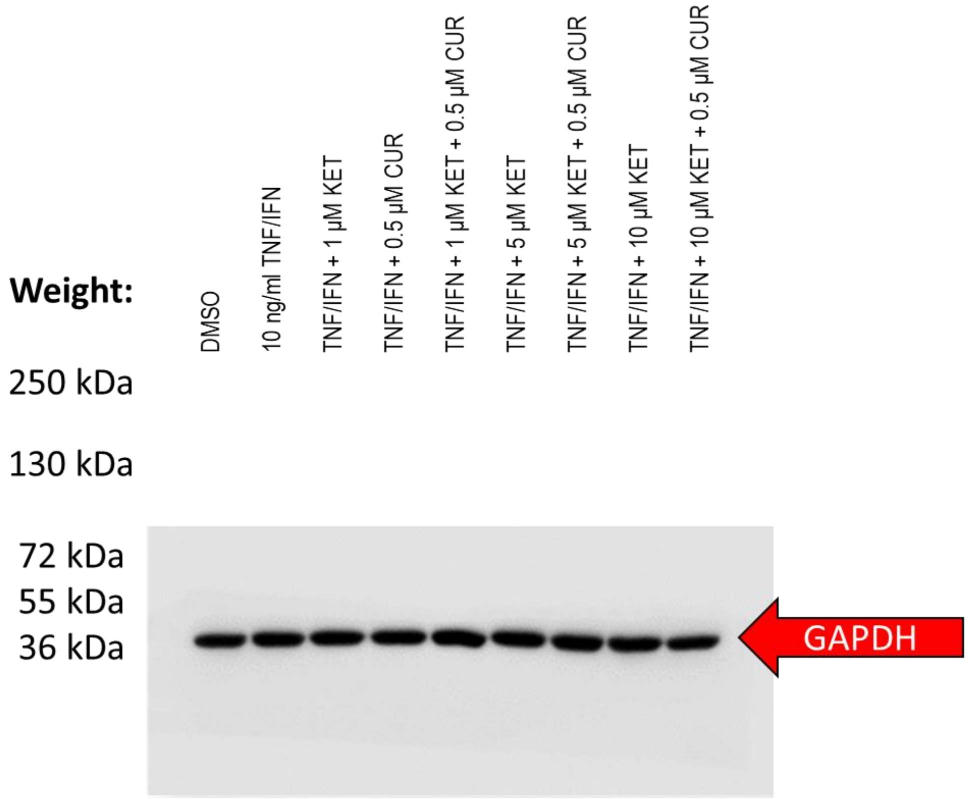

Figure S36. Original Western blots of HSEIC proteins showing GAPDH at molecular weight of 36 kDa. Red arrow indicates bands shown in Figure 14C. This blot membrane has been cut into pieces. Each membrane received different exposure times, and the images were taken separately.

COX-2 (72 kDa) in Figure 15C

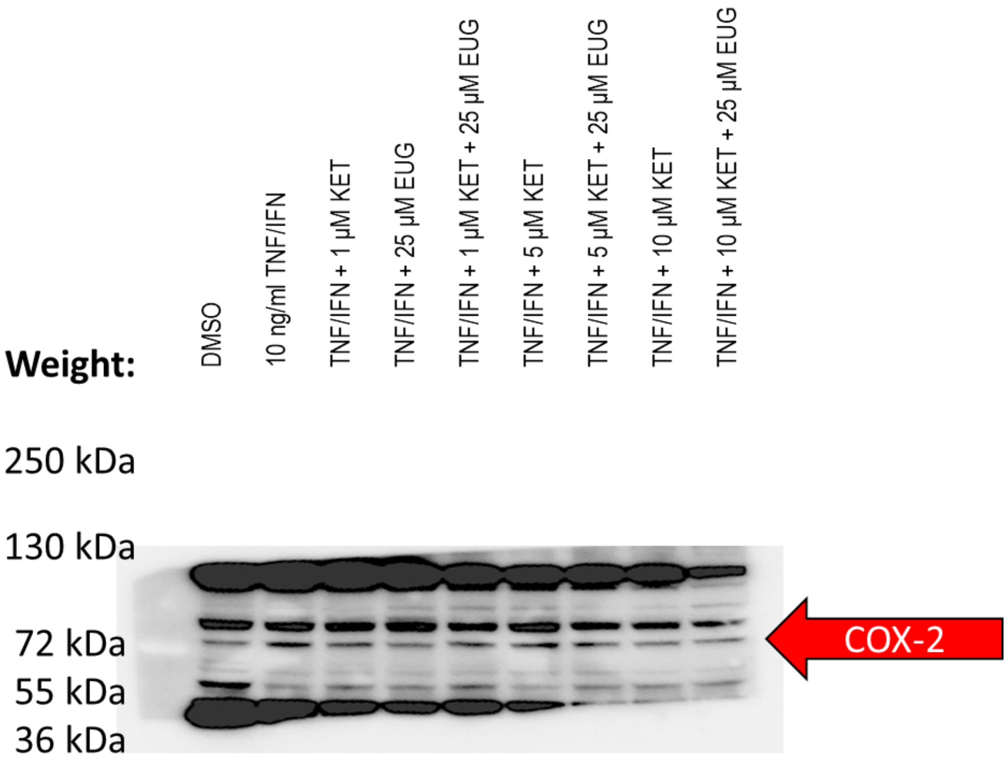

Figure S37. Original Western blots of HSEIC proteins showing COX-2 at molecular weight of 72 kDa. Red arrow indicates bands shown in Figure 15C. This blot membrane has been cut into pieces. Each membrane received different exposure times, and the images were taken separately.

IL-6 (25 kDa) in Figure 15C

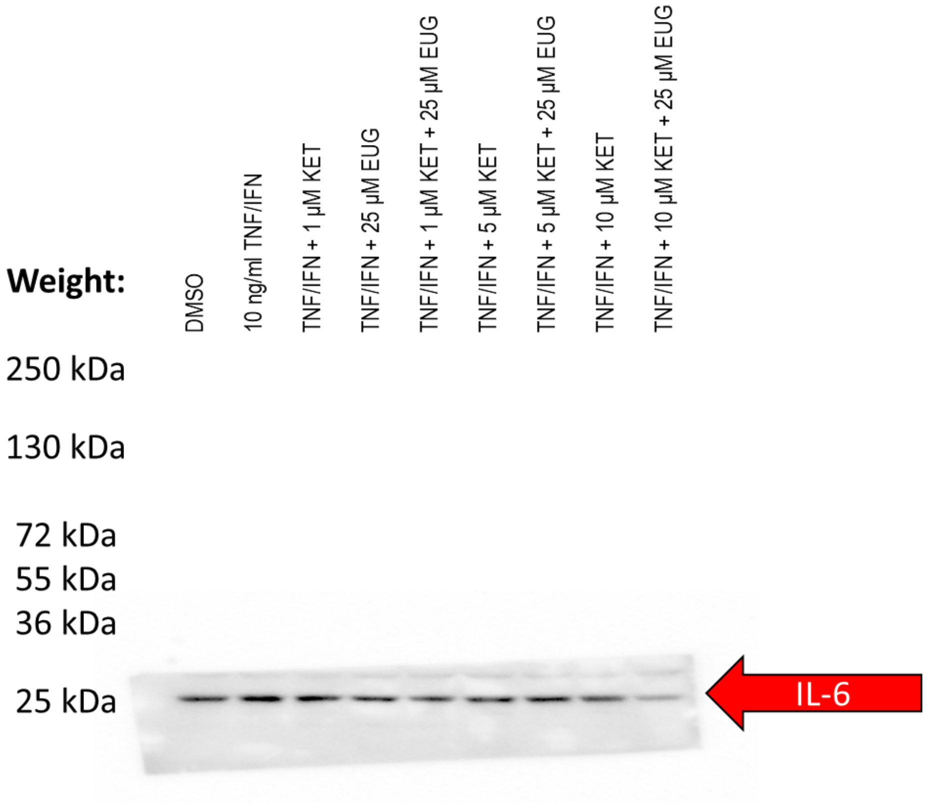

Figure S38. Original Western blots of HSEIC proteins showing IL-6 at molecular weight of 21 kDa. Red arrow indicates bands shown in Figure 15C. This blot membrane has been cut into pieces. Each membrane received different exposure times, and the images were taken separately.

GAPDH (36 kDa) in Figure 15C

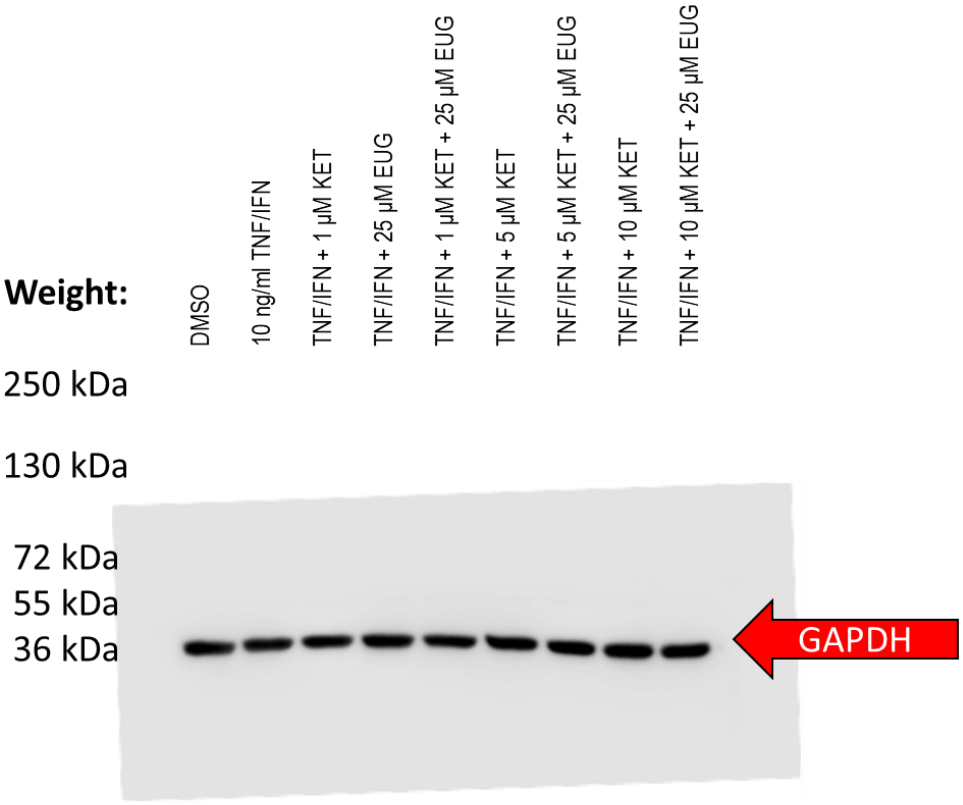

Figure S39. Original Western blots of HSEIC proteins showing GAPDH at molecular weight of 36 kDa. Red arrow indicates bands shown in Figure 15C. This blot membrane has been cut into pieces. Each membrane received different exposure times, and the images were taken separately.
